# Genomic epidemiology of coxsackievirus A16 in mainland of China, 2000–18

**DOI:** 10.1093/ve/veaa084

**Published:** 2020-11-09

**Authors:** Zhenzhi Han, Yang Song, Jinbo Xiao, Lili Jiang, Wei Huang, Haiyan Wei, Jie Li, Hanri Zeng, Qiuli Yu, Jiameng Li, Deshan Yu, Yanjun Zhang, Chonghai Li, Zhifei Zhan, Yonglin Shi, Ying Xiong, Xianjun Wang, Tianjiao Ji, Qian Yang, Shuangli Zhu, Dongmei Yan, Wenbo Xu, Yong Zhang

**Affiliations:** WHO WPRO Regional Polio Reference Laboratory and National Laboratory for Poliomyelitis, NHC Key Laboratory of Biosafety, NHC Key Laboratory of Medical Virology, National Institute for Viral Disease Control and Prevention, Chinese Center for Disease Control and Prevention, No. 155, Changbai Road, Changping District, Beijing, 102206, People’s Republic of China; WHO WPRO Regional Polio Reference Laboratory and National Laboratory for Poliomyelitis, NHC Key Laboratory of Biosafety, NHC Key Laboratory of Medical Virology, National Institute for Viral Disease Control and Prevention, Chinese Center for Disease Control and Prevention, No. 155, Changbai Road, Changping District, Beijing, 102206, People’s Republic of China; WHO WPRO Regional Polio Reference Laboratory and National Laboratory for Poliomyelitis, NHC Key Laboratory of Biosafety, NHC Key Laboratory of Medical Virology, National Institute for Viral Disease Control and Prevention, Chinese Center for Disease Control and Prevention, No. 155, Changbai Road, Changping District, Beijing, 102206, People’s Republic of China; Yunnan Center for Disease Control and Prevention, Kunming, Yunnan Province, People’s Republic of China; Chongqing Center for Disease Control and Prevention, Chongqing City, People’s Republic of China; Henan Center for Disease Control and Prevention, Zhengzhou, Henan Province, People’s Republic of China; Beijing Center for Disease Control and Prevention, Beijing City, People’s Republic of China; Guangdong Center for Disease Control and Prevention, Guangzhou, Guangdong Province, People’s Republic of China; Hebei Center for Disease Control and Prevention, Shijiazhuang, Hebei Province, People’s Republic of China; Tianjin Center for Disease Control and Prevention, Tianjin City, People’s Republic of China; Gansu Center for Disease Control and Prevention, Lanzhou, Gansu Province, People’s Republic of China; Zhejiang Center for Disease Control and Prevention, Hangzhou, Zhejiang Province, People’s Republic of China; Qinghai Center for Disease Control and Prevention, Xining, Qinghai Province, People’s Republic of China; Hunan Center for Disease Control and Prevention, Changsha, Hunan Province, People’s Republic of China; Anhui Center for Disease Control and Prevention, Hefei, Anhui Province, People’s Republic of China; Jiangxi Center for Disease Control and Prevention, Nanchang, Jiangxi Province, People’s Republic of China; Shandong Center for Disease Control and Prevention, Jinan, Shandong Province, People’s Republic of China; WHO WPRO Regional Polio Reference Laboratory and National Laboratory for Poliomyelitis, NHC Key Laboratory of Biosafety, NHC Key Laboratory of Medical Virology, National Institute for Viral Disease Control and Prevention, Chinese Center for Disease Control and Prevention, No. 155, Changbai Road, Changping District, Beijing, 102206, People’s Republic of China; WHO WPRO Regional Polio Reference Laboratory and National Laboratory for Poliomyelitis, NHC Key Laboratory of Biosafety, NHC Key Laboratory of Medical Virology, National Institute for Viral Disease Control and Prevention, Chinese Center for Disease Control and Prevention, No. 155, Changbai Road, Changping District, Beijing, 102206, People’s Republic of China; WHO WPRO Regional Polio Reference Laboratory and National Laboratory for Poliomyelitis, NHC Key Laboratory of Biosafety, NHC Key Laboratory of Medical Virology, National Institute for Viral Disease Control and Prevention, Chinese Center for Disease Control and Prevention, No. 155, Changbai Road, Changping District, Beijing, 102206, People’s Republic of China; WHO WPRO Regional Polio Reference Laboratory and National Laboratory for Poliomyelitis, NHC Key Laboratory of Biosafety, NHC Key Laboratory of Medical Virology, National Institute for Viral Disease Control and Prevention, Chinese Center for Disease Control and Prevention, No. 155, Changbai Road, Changping District, Beijing, 102206, People’s Republic of China; WHO WPRO Regional Polio Reference Laboratory and National Laboratory for Poliomyelitis, NHC Key Laboratory of Biosafety, NHC Key Laboratory of Medical Virology, National Institute for Viral Disease Control and Prevention, Chinese Center for Disease Control and Prevention, No. 155, Changbai Road, Changping District, Beijing, 102206, People’s Republic of China; Center for Biosafety Mega-Science, Chinese Academy of Sciences, Wuhan, Hubei Province, People’s Republic of China; WHO WPRO Regional Polio Reference Laboratory and National Laboratory for Poliomyelitis, NHC Key Laboratory of Biosafety, NHC Key Laboratory of Medical Virology, National Institute for Viral Disease Control and Prevention, Chinese Center for Disease Control and Prevention, No. 155, Changbai Road, Changping District, Beijing, 102206, People’s Republic of China; Center for Biosafety Mega-Science, Chinese Academy of Sciences, Wuhan, Hubei Province, People’s Republic of China

**Keywords:** enterovirus, coxsackievirus A16, phylogeny, epidemiology, phylodynamics

## Abstract

Hand, foot, and mouth disease (HFMD), which is a frequently reported and concerning disease worldwide, is a severe burden on societies globally, especially in the countries of East and Southeast Asia. Coxsackievirus A16 (CV-A16) is one of the most important causes of HFMD and a severe threat to human health, especially in children under 5 years of age. To investigate the epidemiological characteristics, spread dynamics, recombinant forms (RFs), and other features of CV-A16, we leveraged the continuous surveillance data of CV-A16-related HFMD cases collected over an 18-year period. With the advent of the EV-A71 vaccine since 2016, which targeted the EV-A71-related HFMD cases, EV-A71-related HFMD cases decreased dramatically, whereas the CV-A16-related HFMD cases showed an upward trend from 2017 to October 2019. The CV-A16 strains observed in this study were genetically related and widely distributed in the mainland of China. Our results show that three clusters (B1a–B1c) existed in the mainland of China and that the cluster of B1b dominates the diffusion of CV-A16 in China. We found that eastern China played a decisive role in seeding the diffusion of CV-A16 in China, with a more complex and variant transmission trend. Although EV-A71 vaccine was launched in China in 2016, it did not affect the genetic diversity of CV-A16, and its genetic diversity did not decline, which confirmed the epidemiological surveillance trend of CV-A16. Two discontinuous clusters (2000–13 and 2014–18) were observed in the full-length genome and arranged along the time gradient, which revealed the reason why the relative genetic diversity of CV-A16 increased and experienced more complex fluctuation model after 2014. In addition, the switch from RFs B (RF-B) and RF-C co-circulation to RF-D contributes to the prevalence of B1b cluster in China after 2008. The correlation between genotype and RFs partially explained the current prevalence of B1b. This study provides unprecedented full-length genomic sequences of CV-A16 in China, with a wider geographic distribution and a long-term time scale. The study presents valuable information about CV-A16, aimed at developing effective control strategies, as well as a call for a more robust surveillance system, especially in the Asia-Pacific region.

## 1. Introduction

Hand, foot, and mouth disease (HFMD), a frequently reported and concerning disease worldwide, imposes a severe burden on societies globally, especially in the countries of East and Southeast Asia (Xing et al. 2014). HFMD is a mucocutaneous disease that usually causes rashes on the hands, feet, mouth, and buttocks or ulcers and vesicles in the mouth with or without fever. The infectious disease frequently affects children under 5 years of age and is associated with collective outbreaks in schools, parks, and other locations (Zhang and Xu 2013; Xing et al. 2014). In 1997, the largest outbreak of HFMD, which resulted in fatal cases, was reported in Malaysia (Lum et al. 1998). Since then, HFMD outbreaks have been regularly reported in East and Southeast Asia, including the mainland of China (Zhang et al. 2010c). Among patients with HFMD, some develop neurological and systemic complications, including severe neurological symptoms and cardiopulmonary outcomes, which are usually fatal (Ooi et al. 2010).

HFMD consists of an extensive spectrum of viruses of the enterovirus (EV) genus (Xing et al. 2014). In general, EV A71 (EV-A71) and coxsackievirus A16 (CV-A16) are the two major pathogens responsible for HFMD outbreaks (Zhang et al. 2010a, 2011; Xing et al. 2014; Puenpa et al. 2018; Ji et al. 2019b). However, CV-A6, CV-A10, and CV-A4 are common viruses isolated and detected in HFMD cases, which play an important role in the outbreak of HFMD in the world (Hu et al. 2011; Tian et al. 2014; Mirand et al. 2016; Song et al. 2017b; Wang et al. 2018b). Other serotypes of EV, including coxsackievirus B5, coxsackievirus B3, coxsackievirus A8, and other serotypes, have been explored to understand their association with HFMD outbreaks, which have aided in the understanding and control of HFMD (Han et al. 2012, 2019; Liu et al. 2014;  Chen et al. 2016, 2020). Although severe and fatal cases of HFMD are usually linked to EV-A71, CV-A16 has been related to outbreaks of HFMD as well as to severe and fatal cases (Zhang et al. 2010a; Xing et al. 2014; Geoghegan et al. 2015; Takahashi et al. 2016; Wang et al. 2018a).

CV-A16, which was first isolated in South Africa in 1951, has been reported as a major pathogen responsible for many HFMD outbreaks, especially in the Asia-Pacific region; it sometimes causes aseptic meningitis, encephalitis, and myocarditis (Xing et al. 2014; Zell et al. 2017). The first outbreak of HFMD caused by CV-A16 was recorded in Toronto in 1957 (Robinson, Doane, and Rhodes 1958). Since the 1990s, several HFMD outbreaks caused by CV-A16 infection have been reported in Australia, England, Taiwan, Singapore, Vietnam, and India (Ferson and Bell 1991; Van Tu et al. 2007; Chang 2008). Several studies have provided basic data about the clinical symptoms and epidemiological roles of HFMD caused by CV-A16. For example, a large-scale epidemiological study revealed the age distribution, geographic, and seasonal differences of HFMD, which provided the key aspects of major population and temporal scale (Xing et al. 2014; Bubba et al. 2020).

In the mainland of China, CV-A16 was reported as the predominant pathogen responsible for HFMD outbreaks in Beijing in 2007 and Guangzhou in 2009 (Zou et al. 2012; Zhu et al. 2013). Other cities, such as Ningbo, Shenzhen, Huizhou, Shenyang, and Nanchang, also experienced HFMD outbreaks, but CV-A16 was not the dominant cause (Mao et al. 2014). Through the molecular epidemiological analysis of CV-A16 in mainland of China in the past 1999–2008, we can infer the long-term prevalence of CV-A16-related diseases and understand its epidemic laws in order to better control the CV-A16-related diseases (Zhang et al. 2010a). However, recent research on CV-A16 mainly focused on some local regions or cities in China and, therefore, does not represent the wide spread of CV-A16 in the mainland of China. In addition, many molecular epidemiological analyses based on the partial coding regions of CV-A16 improved the understanding of the molecular evolution characteristics of CV-A16 but lacked more comprehensive information on the full-length genome of the virus.

In this study, CV-A16 samples from the mainland of China, covering 22 provinces, were analyzed in depth, including 166 new full-length genomic sequences of CV-A16 collected from 2016 to 2018. In addition, based on the full-length genome of CV-A16, the epidemiological characteristics, the evolutionary dynamics, phylogeography, recombination events, etc., were analyzed. This study provides a comprehensive report of CV-A16, involving the full-length genomic sequences, covering a large area of China, providing an unprecedented perspective to understand the evolution of CV-A16. In addition, this study provides further insight into the epidemiological patterns and evolutionary history of CV-A16 in China, which has guiding significance for the disease control and vaccine evaluation of CV-A16.

## 2. Results

### 2.1 The Persistent Surveillance of HFMD in the Mainland of China

From 2008 to date, both the HFMD case surveillance system and pathogen surveillance laboratory net have been operating for more than 10 years (Zhang et al. 2010c; Xing et al. 2014). The effect of the HFMD case surveillance platform is significant as it dynamically monitors and traces HFMD transmission in the mainland of China for public health. In this study, the epidemiological data of the HFMD surveillance system were scanned and the circulating tendency of HFMD was summarized. The two major pathogens causing HFMD are EV-A71 and CV-A16, although other serotypes of EV can also cause HFMD in China. However, after the application of EV-A71 vaccine, the pathogen spectrum of HFMD in China changed to some extent, and the number of HFMD caused by CV-A6 gradually increased.

**Figure 1.**
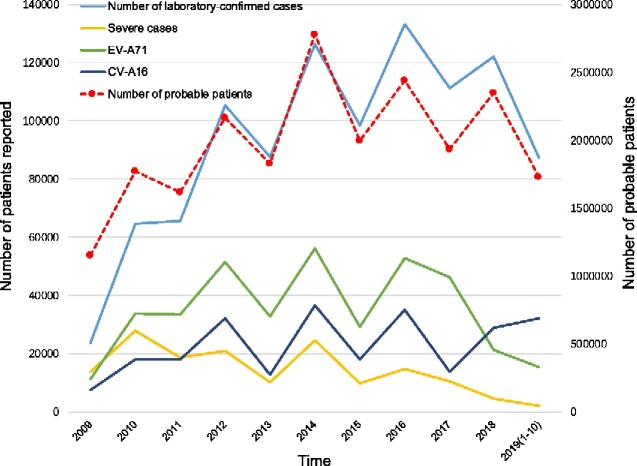
The numbers of probable cases, laboratory-confirmed cases, severe cases, and two major pathogens reported. The dashed line chart shows the sentinel surveillance data of probable HFMD cases reported from 2009 to October 2019, corresponding to the right vertical coordinates. The solid line chart represents the laboratory-confirmed cases, severe cases, EV-A71, and CV-A16, respectively, corresponding to the left vertical coordinates.

The number of probable HFMD cases fluctuates each year, whereas the laboratory-confirmed cases show a similar fluctuation pattern with a higher peak every 2 years (Fig. 1). Before 2016, the number of patients with HFMD caused by EV-A71 presented a fluctuating pattern, followed by a rapid decrease after 2016. The fluctuation trend of severe cases was similar to that of EV-A71-relative HFMD cases. As the EV-A71 vaccine became available in 2016, the number of EV-A71-relative HFMD cases and severe cases of HFMD decreased dramatically, indicating the protective effect of the EV-A71 vaccine (Mao et al. 2016a,b). However, the number of CV-A16-relative HFMD cases remained high after 2017 (until February 2020, data not shown), though the EV-A71 vaccine was available at this time, illustrating that the EV-A71 vaccine does not protect against CV-A16-related HFMD and that multivalent EV vaccines are essential. Before 2017, the number of CV-A16-relative HFMD cases presented a fluctuation pattern similar to the number of probable cases and laboratory-confirmed cases. The increasing number of CV-A16-relative HFMD cases and the dramatically decreasing number of EV-A71-relative HFMD cases revealed the possible pathogen spectrum switch in the mainland of China after 2016. Further investigations on CV-A16 are essential to understand the circulation pattern and multivalent vaccine development of HFMD.

### 2.2 The genomic characteristics, genotyping, and genetic diversity of CV-A16

**Figure 2.**
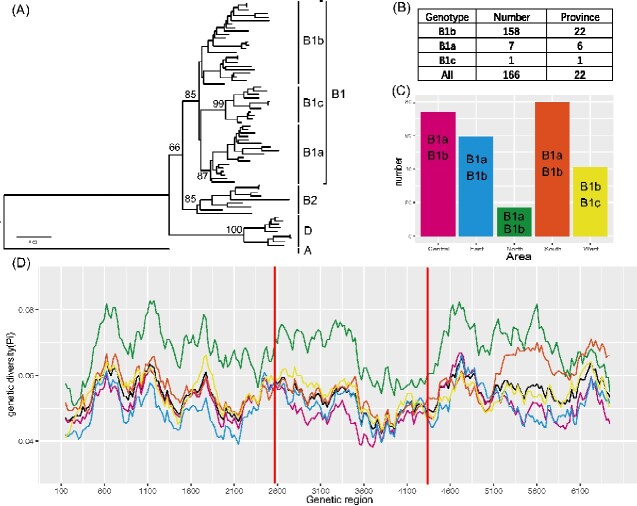
The genetic diversity and statistic numbers of CV-A16 in this study. (A) The neighbor-joining phylogenetic tree based on the entire *VP1* genome of seventy-one representative strains for genotyping. The numbers at each node show the bootstrap values constructed using the neighbor-joining method with 1,000 bootstrap replicates. (B) The numbers and geographic distributions of the CV-A16 sub-genogroups in this study (*n* = 166). (C) The bar chart showing the CV-A16 sample distributions of all representative full-length genomes across five geographic areas in China (*n* = 271). The corresponding genotypes, which were detected in China, are labeled in the bar plot. (D) The genetic diversity of the open reading frame (*ORF)* genome of CV-A16 in this study, which was calculated using a sliding window of 300 nucleotides with a step size of twenty-five nucleotides. The red lines show the partitive genomic region of *P1*, *P2*, and *P3*, respectively. The colored lines represent the corresponding areas, as shown in Fig. 2C.

The representative full-length genomic sequences of CV-A16, which were selected and sequenced during past HFMD pathogen surveillance, were used to investigate the genetic diversity and characteristics. The neighbor-joining phylogenetic tree using the entire *VP1* coding region showed that three genotypes of CV-A16 exist (Fig. 2A). Genotype A consists of a single strain isolated in South Africa in 1951 (strain G10), whereas Genotype B persistently evolved worldwide and was divided into two sub-genogroups with 12 per cent genetic differences between one another. Genotype D, a new genotype of CV-A16, showed 16.7, 16.2, and 31.2 per cent genetic difference compared with sub-genogroup B1, sub-genogroup B2, and Genotype A, respectively. The genetic differences within each genogroup, including Genotype D, sub-genogroup B1, and sub-genogroup B2, were 4.7, 7.4, and 7.5 per cent, respectively. Sub-genogroup B1 was further divided into three clusters designated as B1a, B1b, and B1c, respectively, with 7.8–9.3 per cent genetic diversity among them.

In total, 271 full-length genomic sequences of CV-A16 from the mainland of China were compared with investigate the persistent epidemic and evolution of a dataset of CV-A16 (this study, *n* = 166; GenBank, *n* = 105). Three clusters (B1a–B1c) were found to exist and circulate in the mainland of China, and Cluster B1b still dominates the transmission of CV-A16 here, which is consistent with previous reports (Zhang et al. 2010a; Fig. 2B). The genomic sequences of CV-A16 in this study covered twenty-two provinces of China, forming five geographic regions (Fig. 2C). The B1c strain was found in only one province, and the B1a strains were distributed in six provinces of China, revealing the potential existence of some rare genotypes in the mainland of China (Fig. 2B). Additionally, the ORF genomic sequences of CV-A16 this study shared 85.9–100 per cent nucleotide identity and 95.8–100 per cent amino acid identity among themselves. The nucleotide diversity of the polyprotein showed a tendency of fluctuation, whereas the *VP1* and *2C* coding regions had lower diversity (Fig. 2D).

### 2.3 Evolutionary history and molecular epidemiological analysis of CV-A16 in China

The maximum likelihood phylogenetic tree estimated with a dataset of 271 sequences (this study, *n* = 166; GenBank, *n* = 105) indicated the evolutionary history across the mainland of China between 2000 and 2018 (Supplementary Fig. S1). The result showed that the strains of this study were distributed in every branch of the phylogenetic tree and formed two major genogroups. Most strains of this study were located at the large cluster of phylogenetic trees that were constructed using a different CV-A16 genomic coding region and showed similar topology of phylogenetic trees. From the view of the geographic area distribution of the CV-A16 strains, most strains from different regions were densely located at the maximum likelihood phylogenetic tree and were intersected together, which indicated that the CV-A16 strains were closely related in terms of phylogeny and that CV-A16 transmitted extensively in the mainland of China.

**Figure 3.**
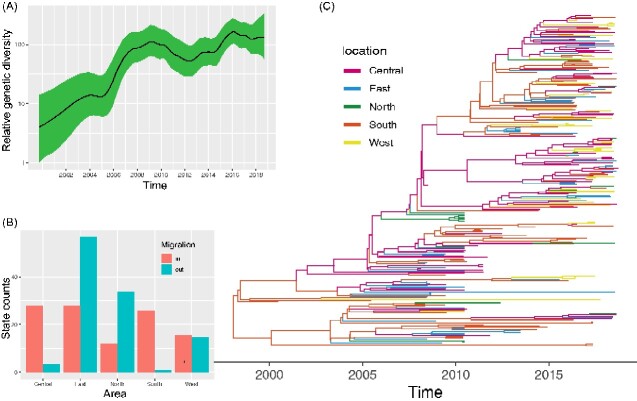
The temporal phylogenies and epidemic characteristics of CV-A16 estimated with the *P1* genomic sequences. (A) The relative genetic diversity of the CV-A16 sequences in China. The x-axis represents the units of year, and the y-axis shows the measure of genetic diversity (logarithmic scale of Neτ, where Ne is the effective population size and τ is the generation time). The black line shows the median estimates of the CV-A16 population size and the green shading represents the 95 per cent CI. (B) The histogram of the average number of state transitions based on five geographic locations. (C) The MCC phylogenetic tree based on the entire *P1* coding region in China and colored according to the different areas.

We performed the Bayesian inference based on the *P1* and *VP1* coding region of CV-A16, because the VP1 coding region was the most external and immunodominant region and P1 coding region coded the capsid protein for EV, which were used for EV typing (19, 28, and 36–38). By Bayesian inference, the Maximum clade credibility (MCC) tree evaluated using a dataset of 271 entire P1 genomic sequences sampled from 2000 to 2018 showed the evolutionary relationships and timescale of CV-A16 (Fig. 3C). The datasets used in the Bayesian analysis passed the date randomization tests, which showed no overlaps between the 95 per cent credibility intervals (CIs) of the rate estimate of real datasets and the 95 per cent CIs produced from twenty replicates of date randomization. Additionally, the root-to-tip regression result supported the relationships between temporal signals and root-to-tip divergence, with *R*^2^ values of 0.81 and 0.88, respectively (Supplementary Fig. S3). The phylogenetic tree presented a ladder-like appearance, which indicated the wide geographic distribution of CV-A16 (Fig. 3C). The CV-A16 strains, which were continuously sampled from 2000 to 2018, were persistently circulated in most locations in China. The strains isolated in the early period (2000–13) were mostly distributed at locations of the ancestral root of the MCC tree (Fig. 3C). Most strains filled the new branches of the phylogenetic tree that gradually evolved from early lineage, which revealed that the CV-A16 strains currently circulating in the mainland of China originated from the propagating strains of the early period. The performance of the MCC tree presented a typical circulation of multiple lineages over time, and the MCC tree using the *VP1* coding region showed identical topology and strain distributions (Supplementary Fig. S2).

### 2.4 Evolutionary characteristics of CV-A16 in China

To assess the evolutionary features of the CV-A16 major genogroups circulating in China, the substation rate, nucleotide divergence, and the their most recent common ancestors (tMRCA) were estimated using a Bayesian relaxed molecular clock. The evolutionary rate of the *VP1* sequence, which was calculated for genogroup B around the world and in the mainland of China, was 3.36 × 10^−3^ [95% (highest probability density) HPD, 2.99–3.74] and 3.74 × 10^−3^ (95% HPD, 3.25–4.27) substitutions per site per year, respectively (Table 1). Additionally, the substitution rate of different sub-genogroups around the world and in the mainland of China ranged from 3.67 × 10^−3^ (95% HPD, 3.13–4.23) to 5.28 × 10^−3^ (95% HPD, 2.99–7.65) substitutions per site per year, respectively. The evolutionary rate of sub-genogroup B1a [5.28 × 10^−3^ (95% HPD, 2.99–7.65) substitutions per site per year] was larger than that of sub-genogroup B1b [4.21 × 10^−3^ (95% HPD, 3.34–5.13) substitutions per site per year] in each dataset. Via the estimated molecular clock, the common ancestor of genogroup B around the world was dated to about 1989 (95% HPD, 1980–94), whereas the tMRCA of genogroup B in the mainland of China was dated to 1994 (95% HPD, 1986–99), which was similar to that reported by others for genogroup B1 that is, 1992 (95% HPD, 1990–94; Hassel et al. 2017). The tMRCA of the different sub-genogroups around the world and in the mainland of China ranged from 1993 (95% HPD, 1987–96) to 2002 (95% HPD, 1991–2007). Moreover, the common ancestor of genogroup B or the sub-genogroups of genogroup B originating from the mainland of China was present later than that of others around the world. The nucleotide divergence and non-synonymous substitution to synonymous substitution (dN/dS) of all sub-genogroups of genogroup B were similar to each other. According to the maximum likelihood phylogenetic tree constructed, no evidence was found regarding geographic origin. Owing to the extensive transmission of CV-A16 to all regions, a geographic cluster for CV-A16 is inapparent (Supplementary Fig. S1). The association index (AI) and parsimony score (PS) value with a significance level <0.001 showed that the CV-A16 *P1* genomic sequences of partial regions were more phylogenetically clustered by region (Table 2). Except for the central region of China, the maximum monophyletic clade (MC) of the other regions presented high values (*P* < 0.05), which indicated that the geographic structure of CV-A16 was significant when the strains were grouped by geographical origin. The isolates of the central region of China did not show a strong geographic cluster (*P* > 0.05), which was possibly caused by the frequent movement of CV-A16. The phenomenon was partially verified by the observed state changes of the central region, with greater imported migration compared with export in this region (Fig. 3B).

**Table 1.** The summary of tMRCA by Markov Chain Monte Carlo (MCMC) analysis, nucleotide divergence, and other parameters.

Genotypes/RFs aNA geographic set	Genotype or RF	*n* ^a^	Divergence^b^				dN/dS^c^		MCMC(BEAST)^d^			
			Nucleotide		Amino acid				Substitution rate (10^−3^) (95% HPD)		tMRCA (95% HPD)	
			VP1	3Dpol	VP1	3Dpol	VP1	3Dpol	VP1	3Dpol	VP1	3Dpol
Whole dataset												
Mainland China	ALL	271	0.065	NA^e^	0.007	NA	0.013	NA	3.74 (3.25–4.27)	NA	1994 (1986–99)	NA
Individual genotype												
All	Genotype B	329	0.074	NA	0.008	NA	0.011	NA	3.36 (2.99–3.74)	NA	1989 (1980–94)	NA
Mainland China	Genotype B	271	0.065	NA	0.007	NA	0.013	NA	3.74 (3.25–4.27)	NA	1994 (1986–99)	NA
Individual sub-genotypes												
All	Sub-genotype B1a	66	0.060	NA	0.006	NA	0.011	NA	4.21 (3.34–5.13)	NA	1993 (1987–96)	NA
All	Sub-genotype B1b	242	0.053	NA	0.007	NA	0.012	NA	3.67 (3.13–4.23)	NA	1994 (1989–98)	NA
All	Sub-genotype B1c	19	0.047	NA	0.009	NA	0.019	NA	NA	NA	NA	NA
Mainland China	Sub-genotype B1a	36	0.057	NA	0.007	NA	0.011	NA	5.28 (2.99–7.65)	NA	2002 (1991–2007)	NA
Mainland China	Sub-genotype B1b	234	0.052	NA	0.006	NA	0.013	NA	4.11 (3.49-4.72)	NA	1998 (1994–2000)	NA
Individual RF groups												
All	RF-A	5	0.192	0.164	0.057	0.032	0.232	0.033	NA	NA	NA	NA
All	RF-B	66	0.063	0.065	0.005	0.018	0.007	0.027	4.21 (3.24–5.21)	4.15(3.19–5.09)	1993(1985–96)	1994(1986–97)
All	RF-C	27	0.067	0.067	0.012	0.021	0.017	0.031	3.81 (2.39–5.22)	5.33(3.70–6.92)	1993(1983–96)	1995(1989–96)
All	RF-D	234	0.052	0.052	0.007	0.016	0.013	0.031	3.70 (3.17–4.30)	3.80(3.30–4.32)	1999(1993–2003)	1997(1988–2002)
All	RF-E	10	0.047	0.038	0.004	0.007	0.009	0.018	8.69 (3.01 × 10^−5^–18)	5.85(3.91 × 10^−6^–13.5)	2008(1988–2010)	2007(1983–2011)

a The number of sequences analyzed in each dataset.

b Overall mean distances.

c dN/dS ratios for each dataset using the SLAC method with a significance value of *P* < 0.05.

d The value was based on the MCMC analysis of each dataset.

e NA, the data values were not calculated for the 3Dpol region because 3Dpol sequences were not monophyletic or the results were not converged.

**Table 2.** Analysis of the geographic structure of the CV-A16 strains in China.

Statistic	Isolates	Observed mean (95% HPD)	Null mean (95% HPD)	Significance
AI		12.32 (11.61, 13.03)	22.46 (21.03, 24.01)	<0.001^***^
PS		109.14 (107, 111)	152.55 (145.83, 159.20)	<0.001^***^
MC(East)	59	4 (4, 4)	2.43 (2, 3.18)	0.02999997^*^
MC(South)	80	6 (6, 6)	2.92(2.01, 4.13)	0.02999997^*^
MC(Central)	74	3 (3, 3)	2.62 (2, 3.47)	0.33999999
MC(West)	41	4 (4, 4)	1.95 (1.02,2.89)	0.01999998^*^
MC(North)	17	5 (5, 5)	1.37 (1, 2)	0.00999999^**^

AI, association index; PS, parsimony score; MC, maximum monophyletic clade; HPD, highest probability density interval.

Significance thresholds: *0.01 < *P* < 0.05; **0.001 < *P* < 0.01; ***P < 0.001.

### 2.5 Spatial transmission dynamics in China

**Figure 4.**
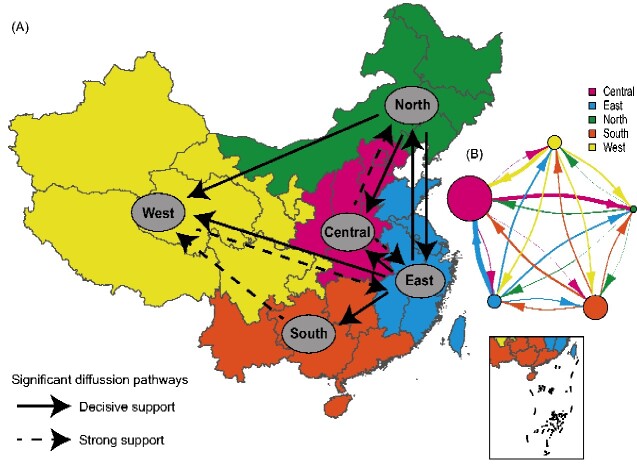
Spatial diffusion of CV-A16 in China. (A) Spatial transmission pathways of CV-A16 inferred using the Bayesian Stochastic Search Variable Selection method. The solid black arrow shows decisive support for the diffusion pathway identified by the *P1* and *VP1* coding regions, with BF > 1,000 and indicator > 0.5. The dashed black arrow represents strong support for the diffusion pathway identified by one of the *P1* and *VP1* coding regions, with BF > 1,000 and indicator > 0.5. (B) The median inferred immigration events identified by the Marginal Structured Coalescent Approximation (MASCOT) method. The width of the arrows indicates the possibility of transmission. The dot sizes are proportional to the median inferred effective population sizes. The five regions of China are defined and colored.

To explore the circulation of CV-A16 in the mainland of China, we constructed past spatial transmission patterns inferred by five geographical regions (Central, South, North, West, and East). In our results, seven decisive migration pathways were identified with strong Bayes factor (BF) and posterior probability (PP) support (BF >1,000 and PP > 0.5). The results revealed that the East region of China plays a decisive role in seeding the CV-A16 epidemics, followed by the North region (Figs 3B, 4A, and Supplementary Table S1). The migrations from East China to all other regions were confirmed through high BF and PP values (PP = 1 and BF > 10,000), whereas the mean migration rates were different for each movement (Supplementary Table S1). The North region of China tended to seed the CV-A16 populations along with human movement. The state counts inferred through Markov jumps method reflected that the outward migration from the East region dominated, which was consistent with the migration pathways (Fig. 3B). The Central, East, and South regions of China showed more or less equal numbers of inward migration, whereas the state counts were similar in terms of the numbers of outward and inward migrations in the West region. Markov rewards values of the Eastern and Northern regions were 356.5 and 381.6, respectively, with a higher proportion compared with those in the other geographic regions, illustrating that these two regions played an important role in the evolution and persistence of CV-A16 over time. Meanwhile, significant links among the five regions were observed with complicated transmission relations. Important spatial diffusion pathways were identified using different inferred methods (Fig. 4B). The results suggested that extensive surveillance in the eastern, central, and northern regions of China, especially in the East, will be helpful for controlling CV-A16 epidemics.

### 2.6 Population dynamics and structure of CV-A16 in China

The reconstruction of the demographic history of CV-A16 in the mainland of China revealed that the CV-A16 population size experienced an oscillating pattern in the relative genetic diversity (Fig. 3A). The results showed that the relative genetic diversity of CV-A16 increased gradually in mainland of China from 2000 to 2018. However, the increasing patterns were divided into three periods; 2000–05, 2005–13, and 2013–18. The virus diversity of the first period expanded, whereas the relative genetic diversity of CV-A16 peaked in 2009 during the second period, with an increasing and then decreasing pattern. The population dynamics of the last period experienced frequent fluctuations during this process. Meanwhile, the EV-A71 vaccine, which has been available in China since 2016, showed substantial influences on EV-A71-related HFMD (Mao et al. 2016a,b). However, the relative genetic diversity of CV-A16 has been stably maintained since 2016, indicating that the EV-A71 vaccine did not influence CV-A16 population dynamics. The phenomenon was consistent with the epidemiological surveillance tendency of CV-A16 (Fig. 1).

**Figure 5.**
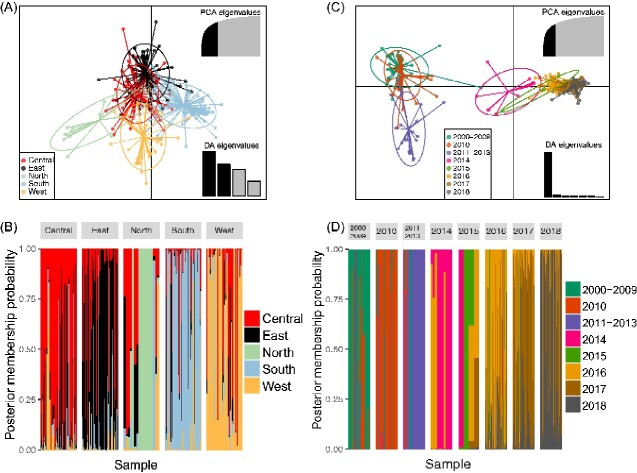
(A) and (C) The scatterplots show the first two principal components of the DAPC of the CV-A16 sequences in mainland China using years of sampling and locations of samples as prior clusters. Eigenvalues of the analysis are displayed in the inset. Groups are shown by different colors and dots representing individual isolates. (B) and (D) Assignment probabilities of CV-A16 individuals from the five sampling locations and years. The panels of CV-A16 individuals are shown according to the years of sampling and locations of samples. The scatterplot was analyzed using the CV-A16 *ORF* coding region.

There was no distinct differentiation among CV-A16 populations based on the *ORF* genome of CV-A16, with these five regions used as prior information (Fig. 5A). As observed in the graphics, the five clusters had significant overlap with each other. Additionally, individuals from the five clusters were found in nearly every region with different relative frequencies (Fig. 5B). However, when the sampling years were used as prior clusters, the marked discontinuity between two successive years, consisting of the clusters of 2000–13 and 2014–18, was confirmed (Fig. 5C). The 2014–18 epidemics were markedly isolated from the other epidemics on the first principal component, revealing that more genetic changes had accumulated during 2013–14 than during the other periods. Moreover, individuals from the cluster of 2014–18 were not found in any other cluster, suggesting that the genetic changes accumulated over time (Fig. 5D). The AI, PS value (*P* < 0.001), and maximum MC (*P* < 0.01) showed that the CV-A16 *P1* genomic sequences were more phylogenetically clustered by two periods (2000–13 and 2014–18), which confirmed the results described above (Supplementary Table S2). The results described above were also found in other discriminant analysis of principal component (DAPC) analysis results based on the CV-A16 *VP1*, *P2*, and *P3* genomic regions, revealing that the accumulation of genetic changes occurred on entire genomes (Supplementary Fig. S4). Additional analyses on the cluster of 2014–18 showed that CV-A16 was genetically structured into different clusters that were arranged along a temporal cline (Supplementary Fig. S5).

### 2.7 Natural selection pressure of CV-A16

To estimate the role of natural selection pressure in different regions or timescale periods, the mixed effects model of evolution (MEME) and single likelihood ancestor counting (SLAC) methods were used. Elevated dN/dS values were observed in the East and Central regions compared with those in the other regions, irrespective of what was analyzed according to the *VP1* or *P1* genomic regions (Supplementary Table S3). The dN/dS values were also higher during 2000–13 than during 2014–18, with slightly variant values. Individual sites under positive selection were identified. Two key sites, which were located at amino acids 145 and 248 within the VP1 capsid protein, were detected. These two amino acid sites were embedded within the loop of the VP1 capsid and were exposed externally, which indicated that the positive sites could play an important role in CV-A16 adaptation to new hosts. The purifying selection was identified at most individual sites, which illustrated that most mutations in the *P1* and *VP1* genomic sequences were deleterious and subsequently removed from the CV-A16 population.

### 2.8 Recombinant forms of CV-A16 around the World

The recombinant forms (RFs), which included 346 available full-length genomic sequences of CV-A16, were analyzed. We referred to other RF reports of EV-A71 and CV-A6 and then grouped the clades (McWilliam Leitch et al. 2012; Gaunt et al. 2015). The phylogenetic tree in the *3D* coding region of CV-A16 formed a series of clades, which were named RF-A, RF-B, RF-C, RF-D, and RF-E, respectively (Supplementary Fig. S6). The nucleotide divergence among the genogroups ranged from 10.1 to 26.9 per cent, whereas the nucleotide distance within each genogroup ranged from 3.8 to 6.7 per cent, except for the nucleotide distance within RF-A. The five RFs, which clearly persisted for many years and were widespread, are still globally active (Table 3). RF-B and RF-C, which have circulated globally since the last century, were the two major recombination models responsible for CV-A16 transmission. These two RFs were closely associated with the genotypes of B1a and B1b, which covered several countries and spread extensively. Additionally, RF-B was linked to the genotype of B2, whereas RF-C was associated with B1c. Interestingly, RF-D was only associated with the genotype of B1b, which dominated the epidemics of CV-A16 in the mainland of China. The transition from early co-circulating RF-B and RF-C to RF-D contributed to the epidemics of CV-A16 after 2008. The performance could partially illustrate the reason that the genotype of B1b was a dominating cluster in the mainland of China at present, with RF-D dominating the RFs and contributing to the evolution of CV-A16. RF-A is an ancient RF of the last century and tends not to be present now. RF-E was an emerging RF in France, which facilitated the appearance of Genotype D (Hassel et al. 2017). Interestingly, based on the *VP1* and *3D* coding regions of CV-A16, the tMRCA of each RF was similar, whereas the substitution rate showed differences (Table 1). The results indicated that the *VP1* genomic region was closely associated with the *3D* coding region and that RFs were linked to the formation of genotypes. The emerging RFs, which were casual incidents and rarely occurred, played a key role in the evolution and epidemics of CV-A16.

**Table 3.** Circulation dates and locations around the world of the different RFs of CV-A16.

Genotype	Recombination	Date of RF	Region of RF
A	RF-A	1951, 2008	South Africa, China, and Malaysia
B1a	RF-A	2012	France
	RF-B	1997–2018	Malaysia, Japan, China, France, Australia, Thailand, Korea, Germany, and Hungary
	RF-C	1997–1998	Malaysia, China
B1b	RF-A	2016	China^a^
	RF-B	2010, 2016	China and USA
	RF-C	2000–08	China, Australia, and Germany
	RF-D	2008–18	China, Australia, France, and Thailand
B1c	RF-C	2005–17	Malaysia, Japan, China, France, India, and Australia
B2	RF-B	2017	China
D	RF-E	2011–14	France and China^a^

a Only one CV-A16 strain sequence found to date.

**Figure 6.**
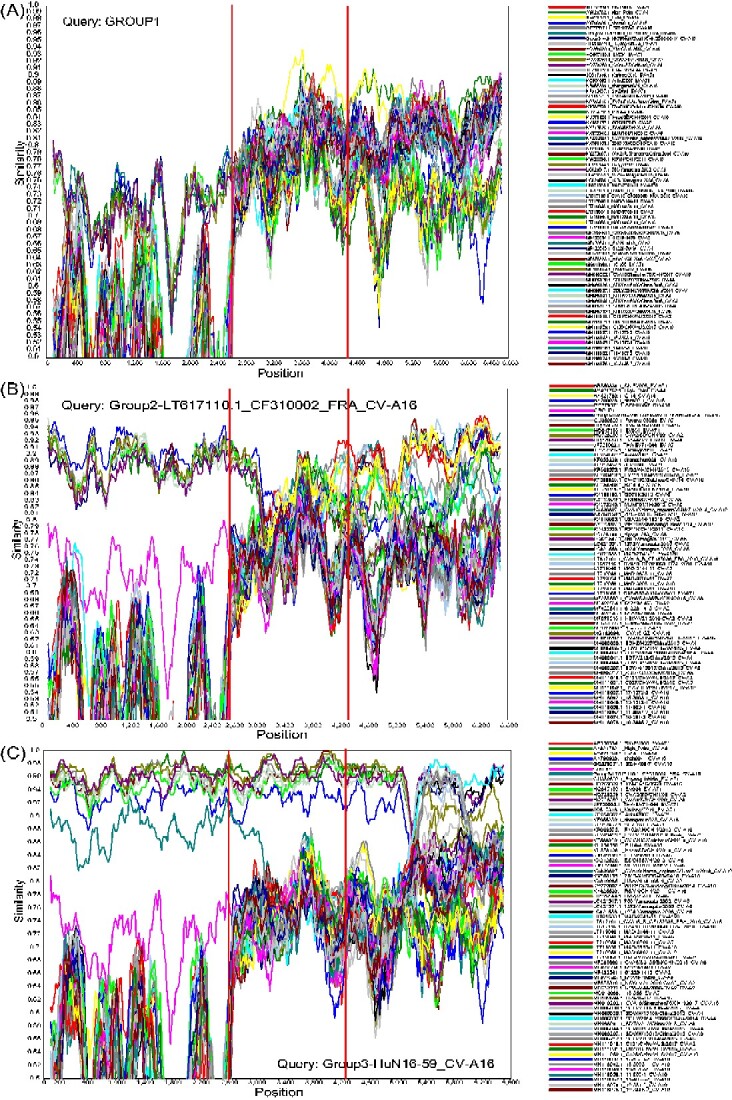
Similarity analysis of the CV-A16 strains with potential parents. The dataset of seventy-two *ORF* genomic sequences, including the representative genomic sequences available from GenBank and this study, was used. The red lines show the partitive genomic region of *P1*, *P2*, and *P3*, respectively. (A) Group 1, including three high identity sequences, was used as a query sequence. (B) Group 2 was used as a query sequence. (C) Group 3 was used as a query sequence.

Previous studies have shown that recombination activities are the general performance for EVs and that EV-B is more susceptible to recombination (Simmonds and Welch 2006; van der Sanden et al. 2011; Fan et al. 2015; Tan et al. 2016; Song et al. 2017a; Han et al. 2018; Huang et al. 2018b; Wang et al. 2018a; Hu et al. 2019). Recombination events of CV-A16, which are recognized as the main mechanism for EV evolution and adaptation to new environments, were occasionally reported compared with other serotypes of EVs such as EV-A71, CV-A6, and CV-A10 (Zhao et al. 2011; Gaunt et al. 2015; Hassel et al. 2017; Noisumdaeng et al. 2018). However, some occasional recombination events were identified in this study (Fig. 6 and Supplementary Fig. S7). Group 1, including three ancient strains with high sequence identity with each other, did not show strong recombination signals with known isolates (Fig. 6A). However, the *P2* genome of Group 1 clustered with the CV-A14, CV-A4, and EV-A71 strains, whereas the *P3* genome of Group 1 clustered with the CV-A4 and CV-A14 strains, exhibiting the complex recombination activities in the evolutionary process of CV-A16 (Supplementary Fig. S7). The *P3* genomic region of Group 2 (GenBank accession number LT617110) showed possible recombination signals with the CV-A6 (GenBank accession number LT719048), CV-A10 (GenBank accession numbers KY272007, KP009575, and KX430807), and CV-A7 (GenBank accession number LT719054) strains, whereas the *P1* coding region had higher similarity with the CV-A16 strain (Fig. 6B and Supplementary Fig. S7). The strain of Group 3, which was first sequenced and reported in this study, showed an extensive recombination event in the *P3* genomic region with the CV-A4 (GenBank accession numbers MH086037, HQ728260, and MF422545), CV-A2 (GenBank accession number HQ728259) and CV-A6 (GenBank accession number LC421551; Fig. 6C) strains. The results revealed that inter-serotype recombination events occasionally occurred in the *P3* coding region of CV-A16 and that most recombination events belonged to intra-serotype recombination. Current circulating CV-A16 strains are recombinants and the exact ancient donors of CV-A16 recombination are unknown, which is consistent with the conclusions of other studies on CV-A16 (Zhao et al. 2011).

## 3. Discussion

The Asia-Pacific region is a major area related to EV-related infectious diseases and is often afflicted by HFMD outbreaks. China is located in the Asia-Pacific region, has a large population, and bears a heavy social burden of HFMD (Huang et al. 2018a). The main pathogens of HFMD are EV-A71 and CV-A16, which usually affect children under 5 years of age and cause collective outbreaks; however, other EVs such as CV-A6, CV-A10, and CV-A4 are increasing rapidly in terms of the proportion of HFMD cases detected. (Tian et al. 2014; Chen et al. 2017; Song et al. 2017b; Anh et al. 2018; Ji et al. 2019a). The national HFMD surveillance system and pathogen laboratory surveillance net were built in the mainland of China in 2008; they play an important role in the response to HFMD outbreaks (Zhang et al. 2010c). The persistent surveillance of HFMD resulted in the accumulation of comprehensive basic data for understanding its epidemiological characteristics, transmission dynamics, and spectrum changes, which provide valuable information for protective countermeasures.

In this study, full-length genomic characteristics, diffusion dynamics, and recombination fragments were analyzed. The continuous surveillance data show that the number of probable HFMD cases and laboratory-confirmed cases experienced similar fluctuations, whereas the performance of EV-A71-related, CV-A16-related, and severe cases was similar before 2016. With the advent of the EV-A71 vaccine in 2016, both the number of patients with EV-A71-related HFMD and the number of severe cases decreased significantly, indicating the beneficial effect of the EV-A71 vaccine. However, the number of HFMD cases related to CV-A16 showed an upward trend from 2017 to October 2019, indicating possible changes in the pathogen spectrum and the demand for multivalent vaccines.

Three genotypes (Genotypes A, B, and D) of CV-A16 exist globally; Genotype B comprises two sub-genogroups. Our results showed that there were three clusters (B1a–B1c) in the mainland of China and that Cluster B1b consistently dominated the diffusion of CV-A16. The results showed that there were still some rare genogroups spread in China. According to the topological structure and distribution of the maximum likelihood phylogenetic tree, the CV-A16 strains were genetically related and were widely distributed in the mainland of China. The ladder-like topological structure of the MCC tree indicated that there were multiple lineages circulating over time; moreover, the current CV-A16 strains may have originated from the early breeding strain. The substitution rate of CV-A6 in China is similar to that in the world, lower than that of EV-A71, CV-A6, and CV-B3 (McWilliam Leitch et al. 2012; Anh et al. 2018; Han et al. 2019). The results showed that the evolution of the CV-A16 *VP1* sequence was slow, which was consistent with the few reports of CV-A16-relative outbreaks in recent years. We found that the common ancestor of Genotype B in the world and in China dated to 1989 (95% HPD, 1980–94) and 1994 (95% HPD, 1986–99), respectively, which was similar to the results reported by other studies (Hassel et al. 2017). Additionally, there were significant differences in the geographical distribution of CV-A16 in China (*P* < 0.05), except for the central region of China, which proves that geography-driven adaptability plays an important role in determining the diversity of CV-A16. However, the interpretation of the results should be cautious.

The spatial transmission relationship of CV-A16 inferred from the five geographic regions of China reveals the past movement pathways of CV-A16, seven of which had decisive migration links. The results show that the East and the North of China, especially the East, played a decisive role in the spread of CV-A16, with higher BF and PP values (PP = 1 and BF > 10,000). In addition, the complex diffusion relationship among the five regions showed a wide variety of transmission trends, consistent with other infectious diseases (Liu et al. 2018). From the perspective of systematic geographic transmission, we suggest that, in addition to the development of a multivalent EV vaccine, extensive and in-depth surveillance in eastern China will help control the prevalence of CV-A16.

The demographic dynamics of CV-A16 showed three oscillation periods. In the past two periods, the relative genetic diversity of CV-A16 peaked in 2009 and 2016, indicating that its dynamics have changed over time. Although the EV-A71 vaccine, which was launched in mainland China in 2016, changed the epidemic characteristics of EV-A71, the relative genetic diversity of CV-A16 did not decrease after 2016. The phenomenon confirms the epidemiological surveillance tendency of CV-A16 and calls for the development of a vaccine related to CV-A16 to control CV-A16-related HFMD. In this study, the population structure of CV-A16 was divided into two discontinuous clusters (2000–13 and 2014–18) based on two successive years, though the previous geographical characteristics were not significant. More genetic changes accumulated during 2013–14, indicating that the population structure in 2014–18 was different from that in 2000–13. Moreover, genetic differences occurred throughout the entire genomes and were arranged along a temporal cline. The result partly explains why the relative genetic diversity of CV-A16 increased since 2014 and presented a more complex fluctuation model during 2013–18 (Fig. 3A).

Elevated dN/dS values were observed in eastern and central China, with higher values in 2000–13 than in 2014–18. Two individual sites, under positive selection pressure, were embedded within the loop of the CV-A16 VP1 capsid, which may play an important role in the adaptation of CV-A16 to new environments.

Five recombination models related to CV-A16 circulation were identified in this study. We found that RF-B and RF-C were closely related to the evolutionary branches B1a and B1b of CV-A16, which were accompanied by the evolution of CV-A16 since the last century. Additionally, RF-D only existed in the genomic sequences of the B1b evolutionary branch and dominated the transmission of CV-A16 in China. In the mainland of China, the switch from RF-B and RF-C co-circulation to RF-D was the reason for the prevalence of the B1b evolutionary branch after 2008. The correlation between genotype and RFs partially explains the current prevalence of the B1b evolutionary branch. The emerging RF-E, a new recombination form, was associated with Genotype D and diffused in European locations (Hassel et al. 2017). Moreover, the tMRCA of each RF, based on the *VP1* and *3D* sequences, respectively, was similar in each dataset. A correlation was observed between the *3D* and *VP1* nucleotide sequences, which proves the necessity of full-length genome analysis and thus provides a comprehensive understanding of CV-A16 evolution. Owing to the lack of genomic sequences of ancient CV-A16, the exact donor of the recombination event of ancient CV-A16 remains unclear. There is evidence that current CV-A16 strains belong to recombinants; however, the exact recombination process is unclear. However, some occasional recombination events were identified in this study, which revealed the form of the inter-serotype recombination. Most of the CV-A16 strains were more likely to occur via intra-serotype recombination than the latter.

This study provides unprecedented full-length genomic sequences of CV-A16 in the mainland of China, covering most provinces of China and spanning a long-term time scale. On the basis of detailed datasets, we estimated and analyzed the evolutionary characteristics, spatial diffusion pathways, novel RFs, and other phylogenetic features of CV-A16 in the mainland of China. The results overcome some limitations of CV-A16 analysis in China. For example, the greater number of full-length genomic sequences and wider sample distribution present a better picture of the evolution process and characteristics of CV-A16. At present, China’s HFMD surveillance system has obvious advantages in controlling HFMD outbreaks, but many countries, especially some countries in the Asia-Pacific region, have not established an HFMD surveillance network. This study demonstrates the importance of HFMD surveillance because the spread of CV-A16 is driven by the movement of infected individuals and CV-A16 distribution is interconnected with different geographical areas. The results show that global HFMD surveillance systems are necessary for the development of effective control strategies, especially in the Asia-Pacific region, which has also been proposed by other studies (Gao 2018; Harvala et al. 2018).

## 4. Methods

### 4.1 Case definitions and the HFMD surveillance system in China

Probable patients with HFMD who had rashes on their hands, feet, mouth, or buttocks and ulcers or vesicles in their mouth with or without fever were defined as patients with HFMD. Laboratory-confirmed patients were identified if there was laboratory evidence of infections with EV-A71, CV-A16, and other EVs. This is consistent with the national regulation principles of HFMD (Xing et al. 2014; Han et al. 2019). After the national HFMD pathogen surveillance net was built in 2008 for mainland China, HFMD cases were reported and representative samples were sent to the national HFMD laboratory for verification. The demographic and clinical features, which included information on the children’s sex, age, and clinical information, were recorded. The raw clinical samples and accompanying information, which were collected by local Centers for Disease Control and Prevention, were reported and submitted to the national HFMD pathogen surveillance lab for the purpose of HFMD surveillance. In addition, the surveillance methods were carried out in accordance with the approved guidelines (Zhang et al. 2010b,c).

### 4.2 Sample collection and viral isolation

The clinical samples were collected successfully for the purpose of public health under the investigation of the National Health Commission of the People’s Republic of China. Moreover, written informed consents for the use of their clinical samples were acquired from the guardians of all children in this study. Simultaneously, the accompanying clinical information, including the children’s sex, age, and disease symptoms, were recorded for the surveillance of EV and public health aims. All clinical samples were processed according to previously described standard procedures and were then inoculated onto human rhabdomyosarcoma cells and human laryngeal epidermoid carcinoma cells provided by the WHO Global Poliovirus Specialized Laboratory for viral isolation (Xu and Zhang 2016; Han et al. 2018). Infected cell cultures were harvested when the complete EV-like cytopathic effect was observed.

### 4.3 Sequencing and molecular typing

Viral RNA was extracted from the cell cultures using the QIAamp Viral RNA Mini Kit (Qiagen, Germany). The diagnosis of CV-A16 was made using real-time quantitative reverse transcription PCR (qRT-PCR) described previously (Zhang et al. 2009; Cui et al. 2013). To amplify the entire *VP1* coding region, we performed RT-PCR using the PrimeScript One Step RT-PCR Kit Ver.2 (TaKaRa, Dalian, China) with primers 486–489 (Oberste et al. 2006). The entire *VP1* region of amplicons was sequenced using an ABI 3130 Genetic Analyzer (Applied Biosystems, Foster City, CA, USA). After the BLAST server and EV Genotyping Tool were used, the *VP1* sequences were analyzed to determine the serotype (Kroneman et al. 2011). The full-length genome sequences were amplified using the ‘primer-walking’ strategy, which gradually closed the gaps of the genome (Zhang et al. 2014). Specific primers of CV-A16 were used (listed in Supplementary Table S4). Additionally, the amplicons produced by RT-PCR products were sequenced on an ABI 3130 Genetic Analyzer (Applied Biosystems, Foster City, CA, USA), as described above.

### 4.4 Selection of representative CV-A16 nucleotide sequences and dataset construction

In total, 1964 CV-A16 strains were identified in the HFMD pathogen surveillance net from 2016 to 2018 in the mainland of China. From these CV-A16 *VP1* datasets, entire *VP1* genome sequences were selected for sequencing and analysis. First, based on the entire *VP1* coding region of CV-A16, we constructed the maximum likelihood phylogenetic tree of different years (2016–18) and randomly selected the representative strains of CV-A16 from each branch of the phylogenetic tree (Importantly, the representative strains should cover more provinces of China for harvesting high representation of geography and cover each cluster of the phylogenetic trees for acquiring a high representation of phylogeny). Thus, in total, 166 clinical samples of CV-A16 were randomly selected for the next procedure, including 53, 50, and 63 samples isolated in 2016, 2017, and 2018, respectively. These samples covered twenty-two provinces of China. Other nucleotide sequences of CV-A16 used in this study were downloaded from the GenBank database. The datasets were filtered by discarding low-quality sequences and non-entire genome sequences. All datasets are listed in Supplementary Table S5.

### 4.5 Phylogenetic analysis

In total, 346 CV-A16 full-length genome sequences in the world, which include 180 full-length genome sequences with known sampling dates and locations from GenBank (dated to August 2019) and 166 full-length genome sequences from this study, were used to analyze the molecular epidemiological characteristics. Moreover, 271 full-length nucleotide sequences of CV-A16, including 166 genome sequences of this study and 105 entire genome sequences from China available from GenBank, were used to analyze the transmission patterns in China. The genome sequences were aligned using the MAFFT software (version v7.407; Katoh and Standley 2013). The maximum likelihood phylogenetic tree was assessed using the IQ-TREE software (version 1.6.1) and inferred using ModelFinder to select the appropriate nucleotide substitution model for different datasets (Nguyen et al. 2015; Kalyaanamoorthy et al. 2017; Zhang et al. 2019).

The genetic diversity and genotyping of CV-A16 groups were assessed using the neighbor-joining method implemented in MEGA (version 7.0.26; Kumar, Stecher, and Tamura 2016). The genetic distance between and within groups was calculated under the kimura 2-parameter model using the bootstrap method. The proportion of nucleotide differences was estimated via pairwise distances (p-distance model) with 1,000 bootstrap replicates.

### 4.6 Phylogeography analysis of CV-A16 and geographic diffusion of CV-A16 strains

Using the Bayesian inference method implemented in the BEAST software (version 1.8.4), the MCC tree and Gaussian Markov random field (GMRF) skyride plots were inferred, with the nucleotide substitution model of SYM+G4 supported by ModelFinder (Drummond et al. 2012; Kalyaanamoorthy et al. 2017). Sampling times of the available sequences were used to calibrate the molecular clock. For each dataset, eighteen individual analyses were run, combined with one nucleotide substitution model, three different clock models, and six different Coalescent tree priors. We performed the analysis using 200 million generations, with sampling every 20,000 generations. Based on the *P1* and *VP1* coding regions, we analyzed the CV-A16 evolutionary dynamics and spatial transmission patterns in the mainland of China. The path sampling and stepping stone sampling analyses, which showed the marginal likelihood estimation results, were implemented in BEAST to choose the best parameters of the Bayesian phylogenetic models (Supplementary Table S6; Baele et al. 2012). The convergence and effective sample size (>200) of the parameters were checked using the Tracer software (version 1.7.1; Drummond et al. 2012). The output trees were summarized using MCC topology from the TreeAnnotator software (version 1.8.4) with a burn-in of the first 10 per cent of sampled trees. The PP values of each node were calculated in the MCC phylogenetic tree. The ggtree (version 1.16.3) was used to manipulate the phylogenetic tree for the best performance (Yu et al. 2017).

Five geographic regions of mainland China (Central region: Beijing, Hebei, Tianjin, Henan, Shanxi, Shanxi, and Hubei provinces; North region: Liaoning, Heilongjiang, Jilin, and Inner Mongolia provinces; South region: Guangdong, Guangxi, Hunan, Yunnan, Guizhou, and Hainan provinces; West region: Sichuan, Gansu, Ningxia, Qinghai, Xinjiang, Chongqing, and Tibet provinces; and East region: Jiangsu, Fujian, Zhejiang, Shanghai, Anhui, Jiangxi, and Shandong provinces) were defined. All genome sequences for Bayesian inference from the five regions were coded as discrete states. The asymmetric substitution model was used to infer the asymmetrical transmission rates between any pairwise region state, including the Bayesian Stochastic Search Variable Selection option (Lemey et al. 2009). The significant migration pathways were retained when BF > 10 and the PP > 0.5. The Markov jumps counts, which estimated the numbers of region state transitions, were used to plot the state counts of migration in and out of each region. In addition, the MASCOT method was also used to estimate the diffusion links among different regions, although it had the precondition of diffusion pathways inference (Muller, Rasmussen, and Stadler 2018). The geographic map of China was drawn from Highcharts (Grant number: 0321912045738052), which was used to display the spread pathways.

To assess the demographic dynamics of CV-A16 in the mainland of China, the coalescent-based GMRF method with the time-aware smoothing parameter was used to construct the past population dynamics (Minin, Bloomquist, and Suchard 2008). The GMRF skyride plots were summarized and visualized using the Tracer software (version 1.7.1).

The randomization tests in the R package (version 3.6.0) using the Tip Dating BEAST package were performed to determine the temporal signal in the data (Rieux and Khatchikian 2017). Using this method, sufficient temporal signals of the datasets are confirmed when the 95 per cent CIs of rate estimation of real datasets do not fall into the 95 per cent CIs of rate estimation from the date-randomized replicates. Based on the TreeTime software, we performed root-to-tip regression analysis, which estimated the relationship between root-to-tip divergence and sample dates (Trovao et al. 2015; Sagulenko, Puller, and Neher 2018). All results support the use of the molecular clock in this study.

### 4.7 Population structure and genetic variation of CV-A16

We defined five populations (Central, North, South, West, and East) of CV-A16 in the mainland of China as described earlier. To determine the extent to which the viral population was constructed by geography, phylogeny-trait association analysis was performed using the BaTS software (version 2.0) to compute the values of the AI, PS, and maximum MC statistics (Parker, Rambaut, and Pybus 2008). *P* values < 0.05 were considered significant from the three statistics. The population structure of CV-A16 in the mainland of China, which was assessed by the DAPC implemented in the adegenet package, was explored (Jombart, Devillard, and Balloux 2010; Jombart and Ahmed 2011). Using the different genome sequences of CV-A16, we inferred the number of clusters of genetically related individuals and analyzed the population structure features of CV-A16 in the mainland of China.

### 4.8 Natural selection and molecular model of CV-A16 *P1* and *VP1* proteins

To compare the selection pressures acting on the *P1* and *VP1* coding regions of CV-A16 in the mainland of China, the MEME method was used to confirm the key selection pressure sites (Murrell et al. 2012). With the significance level of the *P* value set at < 0.05, positively selected sites were identified. We used the SLAC method to assess the ratio of non-synonymous substitution to synonymous substitution (dN/dS) of the different datasets (Kosakovsky Pond and Frost 2005).

### 4.9 Recombination activity of CV-A16 based on the *ORF* coding region

In this study, we scanned the entire genomic sequences of CV-A16 and potential recombinants from GenBank. Briefly, the P2 and P3 coding region sequences of these strains were analyzed using the BLAST server to compare their identity with sequences from GenBank. Sequences with similarity higher than 85 per cent were considered potential parents and were downloaded from GenBank. The Recombinant Detection Program (RDP4, v4.46) was used for primary recombination signal screening of the entire genomic sequences of CV-A16, using seven methods (RDP, GENECONV, MaxChi, Bootscan, Chimaera, SiScan, and 3Seq; Martin et al. 2015). *P* values < 0.05 were considered strong evidence for recombination. Only the reorganization events identified by at least three methods can be considered credible. To confirm these putative recombination events, we utilized a smaller dataset including recombinant and parental strains for multiple screenings. In the end, a dataset of seventy-two full-length genomic sequences of several serotypes was determined for subsequent analysis. The SimPlot software (version 3.5.1) was used for similarity and bootscanning analysis, with a 200-nucleotide sliding window moving in 20-nucleotide steps (Salminen et al. 1995). We inferred recombination breakpoints based on the distribution of informative sites, which supported two incongruent tree topologies that maximized the chi-square sum (Robertson, Hahn, and Sharp 1995).

### 4.10 Ethics statement

Written informed consent for the use of their clinical samples was obtained from all individuals included in the study or their guardians. The study was approved by the Second Ethics Review Committee of the National Institute for Viral Disease Control and Prevention (IVDC), Chinese Center for Disease Control and Prevention; all experimental protocols were approved by IVDC; and the methods were carried out in accordance with the approved guidelines.

## Data availability

The data are available at GenBank (accession numbers: MT211988-MT212036). The genomic data were also uploaded to the China Virus Identification Net (CVIN) under the accession numbers CVIN_AA000315–CVIN_AA000480.

## Authors’ contributions

Z.H., Y.S., and J.X. conceived and performed the experiments. Z.H. analyzed the data, drafted the article, and prepared all the figures. Y.Z. and W.X. conceived and designed the experiments, supervised the project, and revised the article. L.J., W.H., H.W., J.L., H.Z., Q.Y., J.L., D.Y., Y.Z., C.L., Z.Z., Y.S., Y.X., and X.W. collected samples from HFMD patients and performed viral isolation and identification. T.J., Q.Y., S.Z., and D.Y. performed some sequencing work. All authors reviewed the article.

## Supplementary data

Supplementary data are available at *Virus Evolution* online.

## Supplementary Material

veaa084_Supplementary_Data

## References

[veaa084-B1] Anh N. T. et al (2018) ‘ Emerging Coxsackievirus A6 Causing Hand, Foot and Mouth Disease, Vietnam’, Emerging Infectious Diseases, 24: 654–62. 29553326 10.3201/eid2404.171298 PMC5875260

[veaa084-B2] Baele G. et al (2012) ‘ Improving the Accuracy of Demographic and Molecular Clock Model Comparison While Accommodating Phylogenetic Uncertainty’, Molecular Biology and Evolution, 29: 2157–67. 22403239 10.1093/molbev/mss084 PMC3424409

[veaa084-B3] Bubba L. et al (2020) ‘ Circulation of Non-Polio Enteroviruses in 24 EU and EEA Countries between 2015 and 2017: A Retrospective Surveillance Study’, The Lancet Infectious Diseases, 20: 350–61. 31870905 10.1016/S1473-3099(19)30566-3

[veaa084-B4] Chang L. Y. (2008) ‘ Enterovirus 71 in Taiwan’, Pediatrics & Neonatology, 49: 103–12. 19054914 10.1016/S1875-9572(08)60023-6

[veaa084-B5] Chen J. et al (2020) ‘ A Large-Scale Outbreak of Echovirus 30 in Gansu Province of China in 2015 and Its Phylodynamic Characterization’, Frontiers in Microbiology, 11: 1137. 32587581 10.3389/fmicb.2020.01137 PMC7297909

[veaa084-B6] Chen L. et al (2016) ‘ Genomic Characteristics of Coxsackievirus A8 Strains Associated with Hand, Foot, and Mouth Disease and Herpangina’, Archives of Virology, 161: 213–7. 26483280 10.1007/s00705-015-2646-1

[veaa084-B7] Chen M. et al (2017) ‘ Severe Hand, Foot and Mouth Disease Associated with Coxsackievirus A10 Infections in Xiamen, China in 2015’, Journal of Clinical Virology, 93: 20–4. 28577423 10.1016/j.jcv.2017.05.011

[veaa084-B8] Cui A. et al (2013) ‘ The Development and Application of the Two Real-Time RT-PCR Assays to Detect the Pathogen of HFMD’, PLoS One, 8: e61451. 23637836 10.1371/journal.pone.0061451 PMC3630163

[veaa084-B9] Drummond A. J. et al (2012) ‘ Bayesian Phylogenetics with BEAUti and the BEAST 1.7’, Molecular Biology and Evolution, 29: 1969–73. 22367748 10.1093/molbev/mss075 PMC3408070

[veaa084-B10] Fan Q. et al (2015) ‘ A Novel Recombinant Enterovirus Type EV-A89 with Low Epidemic Strength in Xinjiang, China’, Scientific Reports, 5: 18558. 26685900 10.1038/srep18558 PMC4685259

[veaa084-B11] Ferson M. J. Bell S. M. (1991) ‘ Outbreak of Coxsackievirus A16 Hand, Foot, and Mouth Disease in a Child Day-Care Center’, American Journal of Public Health, 81: 1675–6. 1746672 10.2105/ajph.81.12.1675 PMC1405294

[veaa084-B12] Gao G. F. (2018) ‘ From “a”IV to “Z”IKV: Attacks from Emerging and Re-Emerging Pathogens’, Cell, 172: 1157–9. 29522735 10.1016/j.cell.2018.02.025 PMC7126677

[veaa084-B13] Gaunt E. et al (2015) ‘ Genetic Characterization of Human Coxsackievirus A6 Variants Associated with Atypical Hand, Foot and Mouth Disease: A Potential Role of Recombination in Emergence and Pathogenicity’, Journal of General Virology, 96: 1067–79. 25614593 10.1099/vir.0.000062 PMC4631059

[veaa084-B14] Geoghegan J. L. et al (2015) ‘ Phylodynamics of Enterovirus A71-Associated Hand, Foot, and Mouth Disease in Viet Nam’, Journal of Virology, 89: 8871–9. 26085170 10.1128/JVI.00706-15 PMC4524079

[veaa084-B15] Han J. F. et al (2012) ‘ Recombination of Human Coxsackievirus B5 in Hand, Foot, and Mouth Disease Patients, China’, Emerging Infectious Diseases, 18: 351–3. 22305307 10.3201/eid1802.111524 PMC3310474

[veaa084-B16] Han Z. et al (2018) ‘ Genetic Characterization and Molecular Epidemiological Analysis of Novel Enterovirus EV-B80 in China’, Emerging Microbes & Infections, 7: 1–12. 30482903 10.1038/s41426-018-0196-9 PMC6258725

[veaa084-B17] Han Z. et al (2019) ‘ Two Coxsackievirus B3 Outbreaks Associated with Hand, Foot, and Mouth Disease in China and the Evolutionary History Worldwide’, BMC Infectious Diseases, 19: 466. 31126252 10.1186/s12879-019-4107-z PMC6534883

[veaa084-B18] Harvala H. et al (2018) ‘ Recommendations for Enterovirus Diagnostics and Characterisation within and beyond Europe’, Journal of Clinical Virology, 101: 11–7. 29414181 10.1016/j.jcv.2018.01.008

[veaa084-B19] Hassel C. et al (2017) ‘ Phylogeography of Coxsackievirus A16 Reveals Global Transmission Pathways and Recent Emergence and Spread of a Recombinant Genogroup’, Journal of Virology, 91:10.1128/JVI.00630-17 PMC5571250 28659474

[veaa084-B20] Hu L. et al (2019) ‘ Phylogenetic Analysis and Phenotypic Characteristics of Two Tibet EV-C96 Strains’, Virology Journal, 16: 40. 30922336 10.1186/s12985-019-1151-7 PMC6439968

[veaa084-B21] Hu Y. F. et al (2011) ‘ Complete Genome Analysis of Coxsackievirus A2, A4, A5, and A10 Strains Isolated from Hand, Foot, and Mouth Disease Patients in China Revealing Frequent Recombination of Human Enterovirus A’, Journal of Clinical Microbiology, 49: 2426–34. 21543560 10.1128/JCM.00007-11 PMC3147834

[veaa084-B22] Huang J. et al (2018a) ‘ Epidemiology of Recurrent Hand, Foot and Mouth Disease, China, 2008-2015’, Emerging Infectious Diseases, 24: 432–42. 10.3201/eid2403.171303 PMC5823341 29460747

[veaa084-B23] Huang K. et al (2018b) ‘ Antigenic Characteristics and Genomic Analysis of Novel EV-A90 Enteroviruses Isolated in Xinjiang, China’, Scientific Reports, 8: 10247. 29980696 10.1038/s41598-018-28469-9 PMC6035207

[veaa084-B24] Ji T. et al (2019a) ‘ Emerging Recombination of the C2 Sub-Genotype of HFMD-Associated CV-A4 is Persistently and Extensively Circulating in China’, Scientific Reports, 9: 13668. 31541120 10.1038/s41598-019-49859-7 PMC6754396

[veaa084-B25] Ji T. et al (2019b) ‘ Surveillance, Epidemiology, and Pathogen Spectrum of Hand, Foot, and Mouth Disease in Mainland of China from 2008 to 2017’, Biosafety and Health, 1: 32–40.

[veaa084-B26] Jombart T. Ahmed I. (2011) ‘ Adegenet 1.3-1: New Tools for the Analysis of Genome-Wide SNP Data’, Bioinformatics, 27: 3070–1. 21926124 10.1093/bioinformatics/btr521 PMC3198581

[veaa084-B27] Jombart T. Devillard S. Balloux F. (2010) ‘ Discriminant Analysis of Principal Components: A New Method for the Analysis of Genetically Structured Populations’, BMC Genetics, 11: 94. 20950446 10.1186/1471-2156-11-94 PMC2973851

[veaa084-B28] Kalyaanamoorthy S. et al (2017) ‘ ModelFinder: Fast Model Selection for Accurate Phylogenetic Estimates’, Nature Methods, 14: 587–9. 28481363 10.1038/nmeth.4285 PMC5453245

[veaa084-B29] Katoh K. Standley D. M. (2013) ‘ MAFFT Multiple Sequence Alignment Software Version 7: Improvements in Performance and Usability’, Molecular Biology and Evolution, 30: 772–80. 23329690 10.1093/molbev/mst010 PMC3603318

[veaa084-B30] Kosakovsky Pond S. L. Frost S. D. (2005) ‘ Not so Different after All: A Comparison of Methods for Detecting Amino Acid Sites under Selection’, Molecular Biology and Evolution, 22: 1208–22. 15703242 10.1093/molbev/msi105

[veaa084-B31] Kroneman A. et al (2011) ‘ An Automated Genotyping Tool for Enteroviruses and Noroviruses’, Journal of Clinical Virology, 51: 121–5. 21514213 10.1016/j.jcv.2011.03.006

[veaa084-B32] Kumar S. Stecher G. Tamura K. (2016) ‘ MEGA7: Molecular Evolutionary Genetics Analysis Version 7.0 for Bigger Datasets’, Molecular Biology and Evolution, 33: 1870–4. 27004904 10.1093/molbev/msw054 PMC8210823

[veaa084-B33] Lemey P. et al (2009) ‘ Bayesian Phylogeography Finds Its Roots’, PLoS Computational Biology, 5: e1000520. 19779555 10.1371/journal.pcbi.1000520 PMC2740835

[veaa084-B34] Liu Q. et al (2018) ‘ Landscape of Emerging and Re-Emerging Infectious Diseases in China: Impact of Ecology, Climate, and Behavior’, Frontiers of Medicine, 12: 3–22. 29368266 10.1007/s11684-017-0605-9 PMC7089168

[veaa084-B35] Liu X. et al (2014) ‘ Genetic Characterization of Emerging Coxsackievirus A12 Associated with Hand, Foot and Mouth Disease in Qingdao, China’, Archives of Virology, 159: 2497–502. 24796551 10.1007/s00705-014-2067-6

[veaa084-B36] Lum L. C. et al (1998) ‘ Neurogenic Pulmonary Oedema and Enterovirus 71 Encephalomyelitis’, The Lancet, 352: 1391. 10.1016/s0140-6736(05)60789-1 9802304

[veaa084-B37] Mao Q. et al (2016a) ‘ EV-A71 Vaccine Licensure: A First Step for Multivalent Enterovirus Vaccine to Control HFMD and Other Severe Diseases’, Emerging Microbes & Infections, 5: e75. 27436364 10.1038/emi.2016.73 PMC5141264

[veaa084-B38] Mao Q. et al (2014) ‘ Coxsackievirus A16: Epidemiology, Diagnosis, and Vaccine’, Human Vaccines & Immunotherapeutics, 10: 360–7. 24231751 10.4161/hv.27087 PMC4185891

[veaa084-B39] Mao Q. Y. et al (2016b) ‘ EV71 Vaccine, a New Tool to Control Outbreaks of Hand, Foot and Mouth Disease (HFMD)’, Expert Review of Vaccines, 15: 599–606. 26732723 10.1586/14760584.2016.1138862

[veaa084-B40] Martin D. P. et al (2015) ‘ RDP4: Detection and Analysis of Recombination Patterns in Virus Genomes’, Virus Evolution, 1: vev003. 27774277 10.1093/ve/vev003 PMC5014473

[veaa084-B41] Mcwilliam Leitch E. C. et al (2012) ‘ The Association of Recombination Events in the Founding and Emergence of Subgenogroup Evolutionary Lineages of Human Enterovirus 71’, Journal of Virology, 86: 2676–85. 22205739 10.1128/JVI.06065-11 PMC3302253

[veaa084-B42] Minin V. N. Bloomquist E. W. Suchard M. A. (2008) ‘ Smooth Skyride through a Rough Skyline: Bayesian Coalescent-Based Inference of Population Dynamics’, Molecular Biology and Evolution, 25: 1459–71. 18408232 10.1093/molbev/msn090 PMC3302198

[veaa084-B43] Mirand A. et al (2016) ‘ Ambulatory Pediatric Surveillance of Hand, Foot and Mouth Disease as Signal of an Outbreak of Coxsackievirus A6 Infections, France, 2014-2015’, Emerging Infectious Diseases, 22: 1884–93. 27767012 10.3201/eid2211.160590 PMC5088007

[veaa084-B44] Muller N. F. Rasmussen D. Stadler T. (2018) ‘ MASCOT: Parameter and State Inference under the Marginal Structured Coalescent Approximation’, Bioinformatics, 34: 3843–8. 29790921 10.1093/bioinformatics/bty406 PMC6223361

[veaa084-B45] Murrell B. et al (2012) ‘ Detecting Individual Sites Subject to Episodic Diversifying Selection’, PLoS Genetics, 8: e1002764. 22807683 10.1371/journal.pgen.1002764 PMC3395634

[veaa084-B46] Nguyen L. T. et al (2015) ‘ IQ-TREE: A Fast and Effective Stochastic Algorithm for Estimating Maximum-Likelihood Phylogenies’, Molecular Biology and Evolution, 32: 268–74. 25371430 10.1093/molbev/msu300 PMC4271533

[veaa084-B47] Noisumdaeng P. et al (2018) ‘ Complete Genome Analysis Demonstrates Multiple Introductions of Enterovirus 71 and Coxsackievirus A16 Recombinant Strains into Thailand during the past Decade’, Emerging Microbes & Infections, 7: 1–12. 30552334 10.1038/s41426-018-0215-x PMC6294798

[veaa084-B48] Oberste M. S. et al (2006) ‘ Species-Specific RT-PCR Amplification of Human Enteroviruses: A Tool for Rapid Species Identification of Uncharacterized Enteroviruses’, Journal of General Virology, 87: 119–28. 16361424 10.1099/vir.0.81179-0

[veaa084-B49] Ooi M. H. et al (2010) ‘ Clinical Features, Diagnosis, and Management of Enterovirus 71’, The Lancet Neurology, 9: 1097–105. 20965438 10.1016/S1474-4422(10)70209-X

[veaa084-B50] Parker J. Rambaut A. Pybus O. G. (2008) ‘ Correlating Viral Phenotypes with Phylogeny: Accounting for Phylogenetic Uncertainty’, Infection, Genetics and Evolution, 8: 239–46. 10.1016/j.meegid.2007.08.001 17921073

[veaa084-B51] Puenpa J. et al (2018) ‘ Enterovirus A71 Infection, Thailand, 2017’, Emerging Infectious Diseases, 24: 1386–7. 29912701 10.3201/eid2407.171923 PMC6038748

[veaa084-B52] Rieux A. Khatchikian C. E. (2017) ‘ Tipdatingbeast: An r Package to Assist the Implementation of Phylogenetic Tip-Dating Tests Using Beast’, Molecular Ecology Resources, 17: 608–13. 27717245 10.1111/1755-0998.12603

[veaa084-B53] Robertson D. L. Hahn B. H. Sharp P. M. (1995) ‘ Recombination in AIDS Viruses’, Journal of Molecular Evolution, 40: 249–59. 7723052 10.1007/BF00163230

[veaa084-B54] Robinson C. R. Doane F. W. Rhodes A. J. (1958) ‘ Report of an Outbreak of Febrile Illness with Pharyngeal Lesions and Exanthem: Toronto, Summer 1957; Isolation of Group a Coxsackie Virus’, Canadian Medical Association Journal, 79: 615–21. 13585281 PMC1830188

[veaa084-B55] Sagulenko P. Puller V. Neher R. A. (2018) ‘ TreeTime: Maximum-Likelihood Phylodynamic Analysis’, Virus Evolution, 4: vex042. 29340210 10.1093/ve/vex042 PMC5758920

[veaa084-B56] Salminen M. O. et al (1995) ‘ Identification of Breakpoints in Intergenotypic Recombinants of HIV Type 1 by Bootscanning’, AIDS Research and Human Retroviruses, 11: 1423–5. 8573403 10.1089/aid.1995.11.1423

[veaa084-B57] Simmonds P. Welch J. (2006) ‘ Frequency and Dynamics of Recombination within Different Species of Human Enteroviruses’, Journal of Virology, 80: 483–93. 16352572 10.1128/JVI.80.1.483-493.2006 PMC1317522

[veaa084-B58] Song Y. et al (2017a) ‘ Phylogenetic Characterizations of Highly Mutated EV-B106 Recombinants Showing Extensive Genetic Exchanges with Other EV-B in Xinjiang, China’, Scientific Reports, 7: 43080. 28230168 10.1038/srep43080 PMC5322377

[veaa084-B59] Song Y. et al (2017b) ‘ Persistent Circulation of Coxsackievirus A6 of Genotype D3 in Mainland of China between 2008 and 2015’, Scientific Reports, 7: 5491. 28710474 10.1038/s41598-017-05618-0 PMC5511160

[veaa084-B60] Takahashi S. et al (2016) ‘ Hand, Foot, and Mouth Disease in China: Modeling Epidemic Dynamics of Enterovirus Serotypes and Implications for Vaccination’, PLoS Medicine, 13: e1001958. 26882540 10.1371/journal.pmed.1001958 PMC4755668

[veaa084-B61] Tan Y. et al (2016) ‘ Molecular Evolution and Intraclade Recombination of Enterovirus D68 during the 2014 Outbreak in the United States’, Journal of Virology, 90: 1997–2007. 26656685 10.1128/JVI.02418-15 PMC4733988

[veaa084-B62] Tian H. et al (2014) ‘ Prevalence of Multiple Enteroviruses Associated with Hand, Foot, and Mouth Disease in Shijiazhuang City, Hebei Province, China: Outbreaks of Coxsackieviruses a10 and b3’, PLoS One, 9: e84233. 24392117 10.1371/journal.pone.0084233 PMC3879295

[veaa084-B63] Trovao N. S. et al (2015) ‘ Host Ecology Determines the Dispersal Patterns of a Plant Virus’, Virus Evolution, 1: vev016. 27774287 10.1093/ve/vev016 PMC5014491

[veaa084-B64] van der Sanden S. et al (2011) ‘ Detection of Recombination Breakpoints in the Genomes of Human Enterovirus 71 Strains Isolated in The Netherlands in Epidemic and Non-Epidemic Years, 1963-2010’, Infection, Genetics and Evolution, 11: 886–94. 10.1016/j.meegid.2011.02.011 21352955

[veaa084-B65] Van Tu P. et al (2007) ‘ Epidemiologic and Virologic Investigation of Hand, Foot, and Mouth Disease, Southern Vietnam, 2005’, Emerging Infectious Diseases, 13: 1733–41. 18217559 10.3201/eid1311.070632 PMC3375788

[veaa084-B66] Wang J. et al (2018a) ‘ The Emergence and Spread of One Coxsackievirus A16 Genogroup D Novel Recombinant Strain That Caused a Clustering HFMD Outbreak in Shanghai, China, 2016’, Emerging Microbes & Infections, 7: 1–3. 30022051 10.1038/s41426-018-0134-x PMC6052075

[veaa084-B67] Wang S. H. et al (2018b) ‘ Divergent Pathogenic Properties of Circulating Coxsackievirus A6 Associated with Emerging Hand, Foot, and Mouth Disease’, Journal of Virology, 92:10.1128/JVI.00303-18 PMC5952127 29563294

[veaa084-B68] Xing W. et al (2014) ‘ Hand, Foot, and Mouth Disease in China, 2008-12: An Epidemiological Study’, The Lancet Infectious Diseases, 14: 308–18. 24485991 10.1016/S1473-3099(13)70342-6 PMC4035015

[veaa084-B69] Xu W. Zhang Y. (2016) ‘ Isolation and Characterization of Vaccine-Derived Polioviruses, Relevance for the Global Polio Eradication Initiative’, Methods in Molecular Biology (Clifton, N.J.), 1387: 213–26. 10.1007/978-1-4939-3292-4_10 26983736

[veaa084-B70] Yu G. et al (2017) ‘ Ggtree: Anrpackage for Visualization and Annotation of Phylogenetic Trees with Their Covariates and Other Associated Data’, Methods in Ecology and Evolution, 8: 28–36.

[veaa084-B71] Zell R. et al; ICTV Report Consortium. (2017) ‘ ICTV Virus Taxonomy Profile: Picornaviridae’, Journal of General Virology, 98: 2421–2. 28884666 10.1099/jgv.0.000911 PMC5725991

[veaa084-B72] Zhang Y. Xu W. B. (2013) ‘ Molecular Epidemiology of Enteroviruses Associated with Hand, Foot, and Mouth Disease in the Mainland of China’, Biomedical and Environmental Sciences, 26: 875–6. 24331531 10.3967/bes2013.015

[veaa084-B73] Zhang D et al. (2020) ‘ PhyloSuite: An Integrated and Scalable Desktop Platform for Streamlined Molecular Sequence Data Management and Evolutionary Phylogenetics Studies’, *Molecular Ecology Resources*, 20: 348–55. 10.1111/1755-0998.13096 31599058

[veaa084-B74] Zhang Y. et al (2009) ‘ An Outbreak of Hand, Foot, and Mouth Disease Associated with Subgenotype C4 of Human Enterovirus 71 in Shandong, China’, Journal of Clinical Virology, 44: 262–7. 19269888 10.1016/j.jcv.2009.02.002

[veaa084-B75] Zhang Y. et al (2010a) ‘ Molecular Evidence of Persistent Epidemic and Evolution of Subgenotype B1 Coxsackievirus A16-Associated Hand, Foot, and Mouth Disease in China’, Journal of Clinical Microbiology, 48: 619–22. 20018819 10.1128/JCM.02338-09 PMC2815627

[veaa084-B76] Zhang Y. et al (2010b) ‘ Type 2 Vaccine-Derived Poliovirus from Patients with Acute Flaccid Paralysis in china: Current Immunization Strategy Effectively Prevented Its Sustained Transmission’, The Journal of Infectious Diseases, 202: 1780–8. 21050127 10.1086/657410

[veaa084-B77] Zhang Y. et al (2010c) ‘ An Emerging Recombinant Human Enterovirus 71 Responsible for the 2008 Outbreak of Hand Foot and Mouth Disease in Fuyang City of China’, Virology Journal, 7: 94. 20459851 10.1186/1743-422X-7-94 PMC2885340

[veaa084-B78] Zhang Y. et al (2011) ‘ Emergence and Transmission Pathways of Rapidly Evolving Evolutionary Branch C4a Strains of Human Enterovirus 71 in the Central Plain of China’, PLoS One, 6: e27895. 22125635 10.1371/journal.pone.0027895 PMC3220707

[veaa084-B79] Zhang Y. et al (2014) ‘ Molecular Typing and Characterization of a New Serotype of Human Enterovirus (EV-B111) Identified in China’, Virus Research, 183: 75–80. 24503225 10.1016/j.virusres.2014.01.002

[veaa084-B80] Zhao K. et al (2011) ‘ Circulating Coxsackievirus A16 Identified as Recombinant Type a Human Enterovirus, China’, Emerging Infectious Diseases, 17: 1537–40. 21801645 10.3201/eid1708.101719 PMC3381541

[veaa084-B81] Zhu J. et al (2013) ‘ Phylogenetic Analysis of Enterovirus 71 Circulating in Beijing, China from 2007 to 2009’, PLoS One, 8: e56318. 23418551 10.1371/journal.pone.0056318 PMC3572022

[veaa084-B82] Zou X. N. et al (2012) ‘ Etiologic and Epidemiologic Analysis of Hand, Foot, and Mouth Disease in Guangzhou City: A Review of 4,753 Cases’, The Brazilian Journal of Infectious Diseases, 16: 457–65. 22964289 10.1016/j.bjid.2012.08.001

