# Supplementary material for: Genomic epidemiology of coxsackievirus A16 in mainland of China, 2000–18
Source: Virus Evol. 2020 Nov 9;6(2):veaa084. doi: 10.1093/ve/veaa084 (PMC7733612; doi:10.1093/ve/veaa084)
Supplement: veaa084_Supplementary_Data [file veaa084_supplementary_data.pdf]

Supplementary Material

Fig S1. The maximum likelihood phylogenetic tree based on the open reading fragment (*ORF*), *P1*, *P2*, and *P3* coding regions of 271 CV-A16 genomic sequences. The phylogenetic tree branches of strains isolated from this study were colored according to geographic regions. Other CV-A16 sequences from GenBank were colored in grey. The scale bars represent the substitutions per site per year. The strain of AY790926.1/shzh00-1/China/2000 was rooted as an outgroup. (A) *ORF* coding sequences; (B) *P1* coding sequences; (C) *P2* coding sequences; (D) *P3* coding sequences.

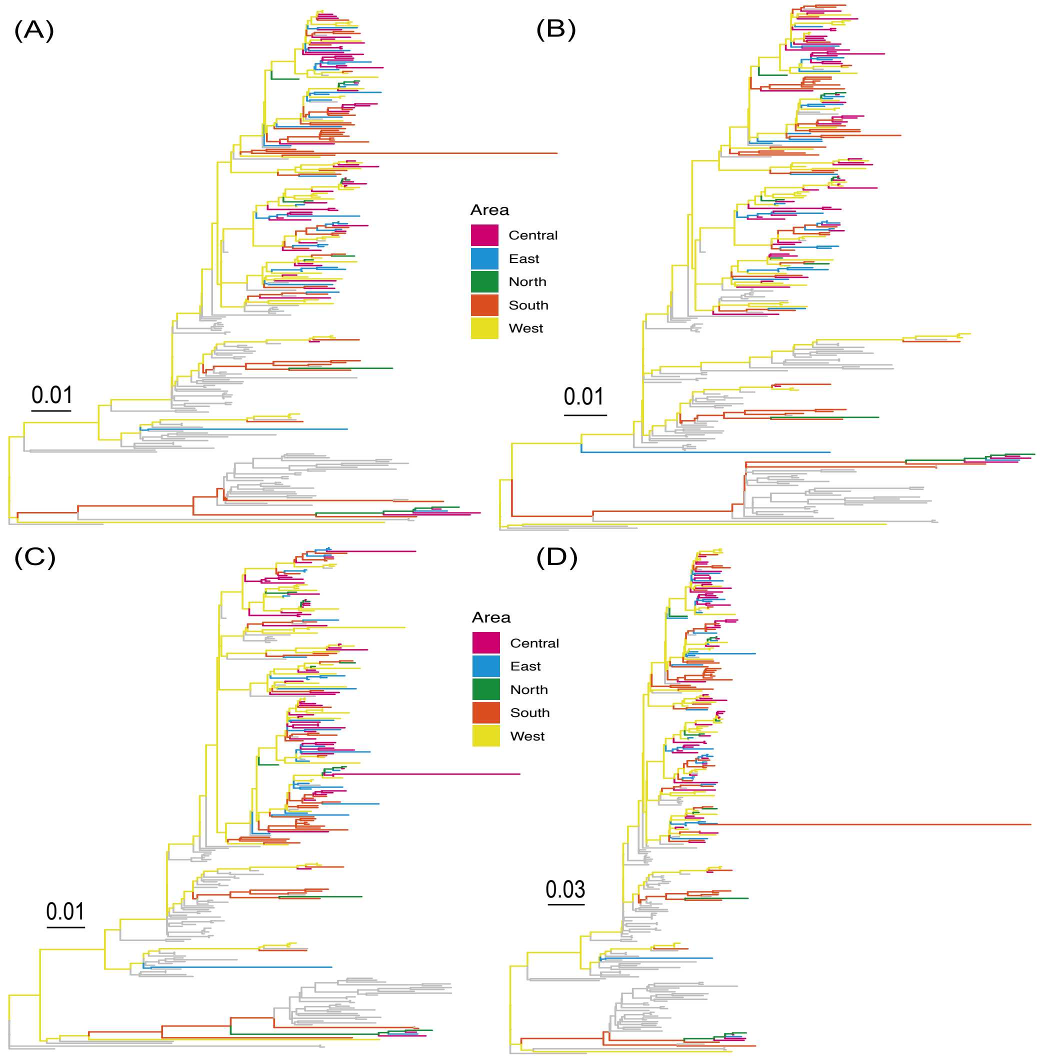

Fig S2. The temporal phylogenies and epidemic characteristics of CV-A16 estimated with the *VP1* genomic sequences. (A) The relative genetic diversity of CV-A16 sequences in China. The x-axis represents the units of year and the y-axis shows the measure of genetic diversity (logarithmic scale of Neτ, where Ne is the effective population size and τ is the generation time). The black line shows the median estimates of CV-A16 population size and the green shading represent 95% credibility internally. (B) The histogram of the average number of state transitions based on five geographic locations. (C) The maximum clade credibility (MCC) phylogenetic tree based on the entire *VP1* coding region in China and colored according to different areas.

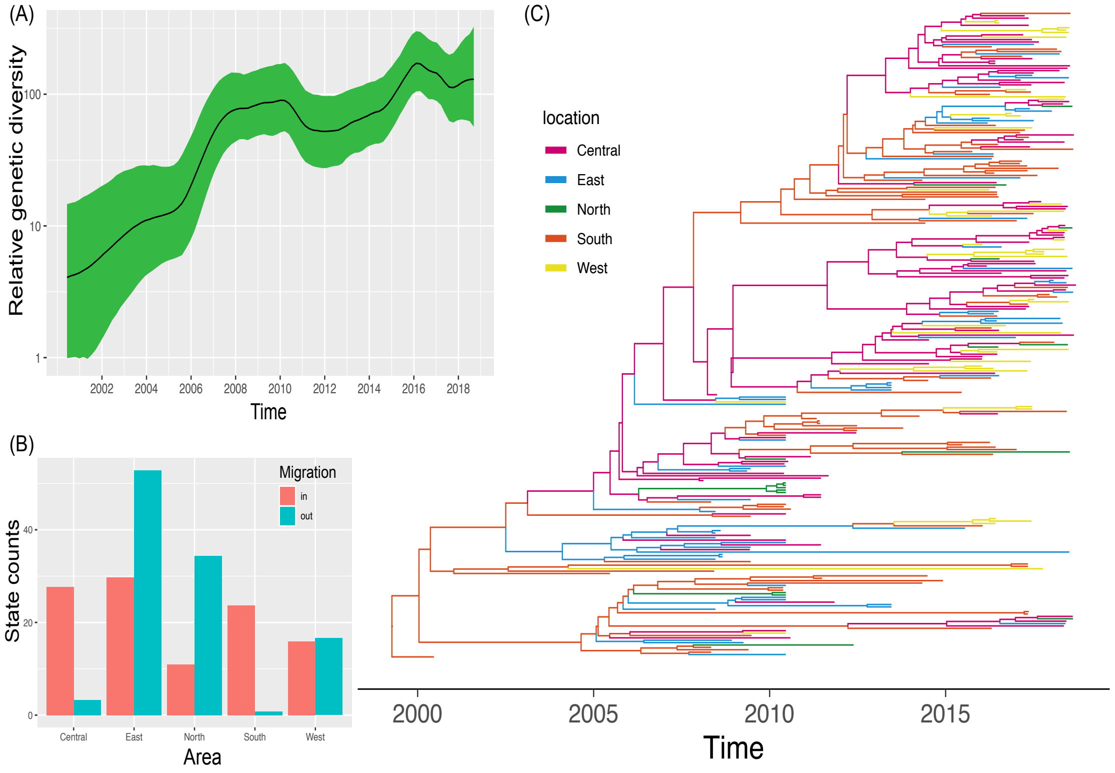

Fig S3. The results of date-randomization tests (DRTs) and root-to-tip regression. (A) Based on the estimation of the *VP1* genomic region of CV-A16, the left panel shows

the result of DRTs and the right panel indicates the linear regression of root-to-tip divergence on dates. (B) Based on the estimation of the *P1* genomic region of CV-A16, the left panel shows the result of DRTs and the right panel indicates the linear regression of root-to-tip divergence on dates. The temporal signal of CV-A16 datasets was estimated using these two methods to assure the sufficient temporal signals for the next assessment.

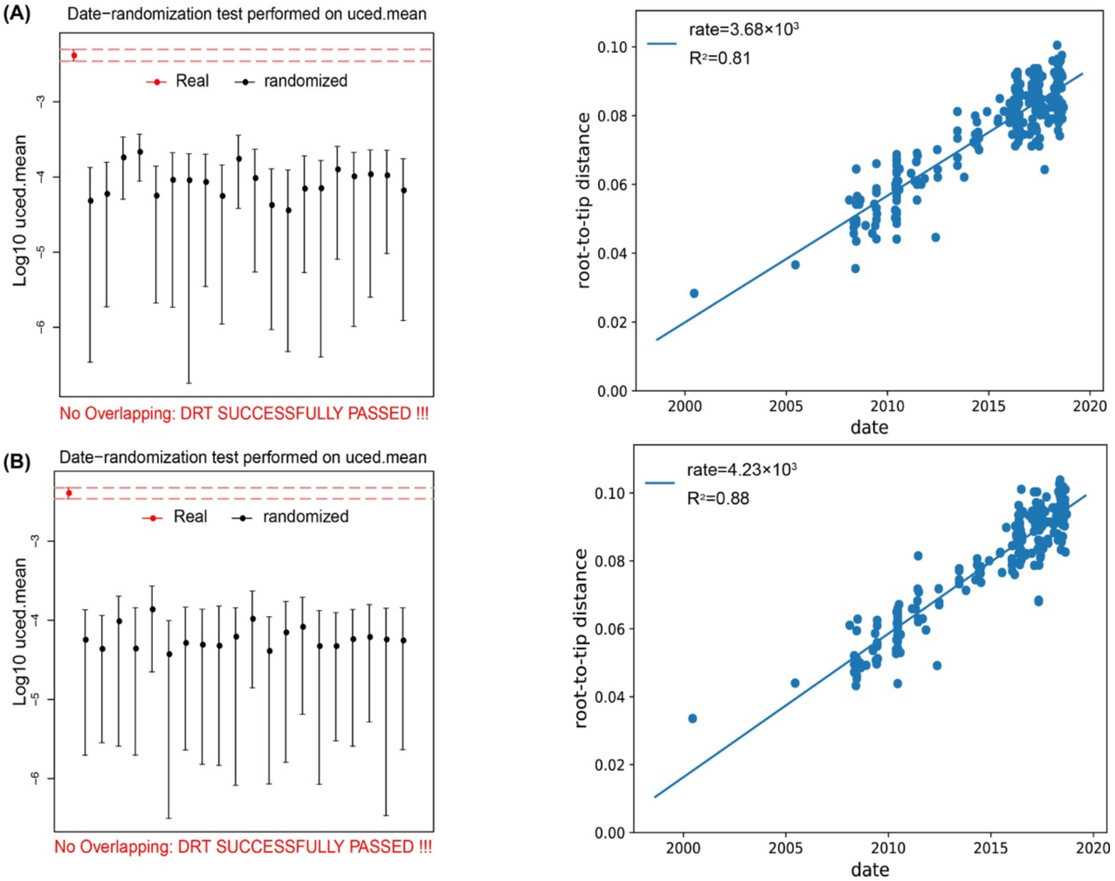

Fig S4. The scatterplots show the first two principal components of the DAPC of CV-A16 sequences in mainland of China, using years of sampling and locations of samples

as prior clusters. Eigenvalues of the analysis are displayed in the inset. Groups are shown by different colors and dots representing individual isolates. (A) The scatterplot was analyzed using the CV-A16 *VP1* coding region. (B) The scatterplot was analyzed using the CV-A16 *P2* coding region. (C) The scatterplot was analyzed using the CV-A16 *P3* coding region.

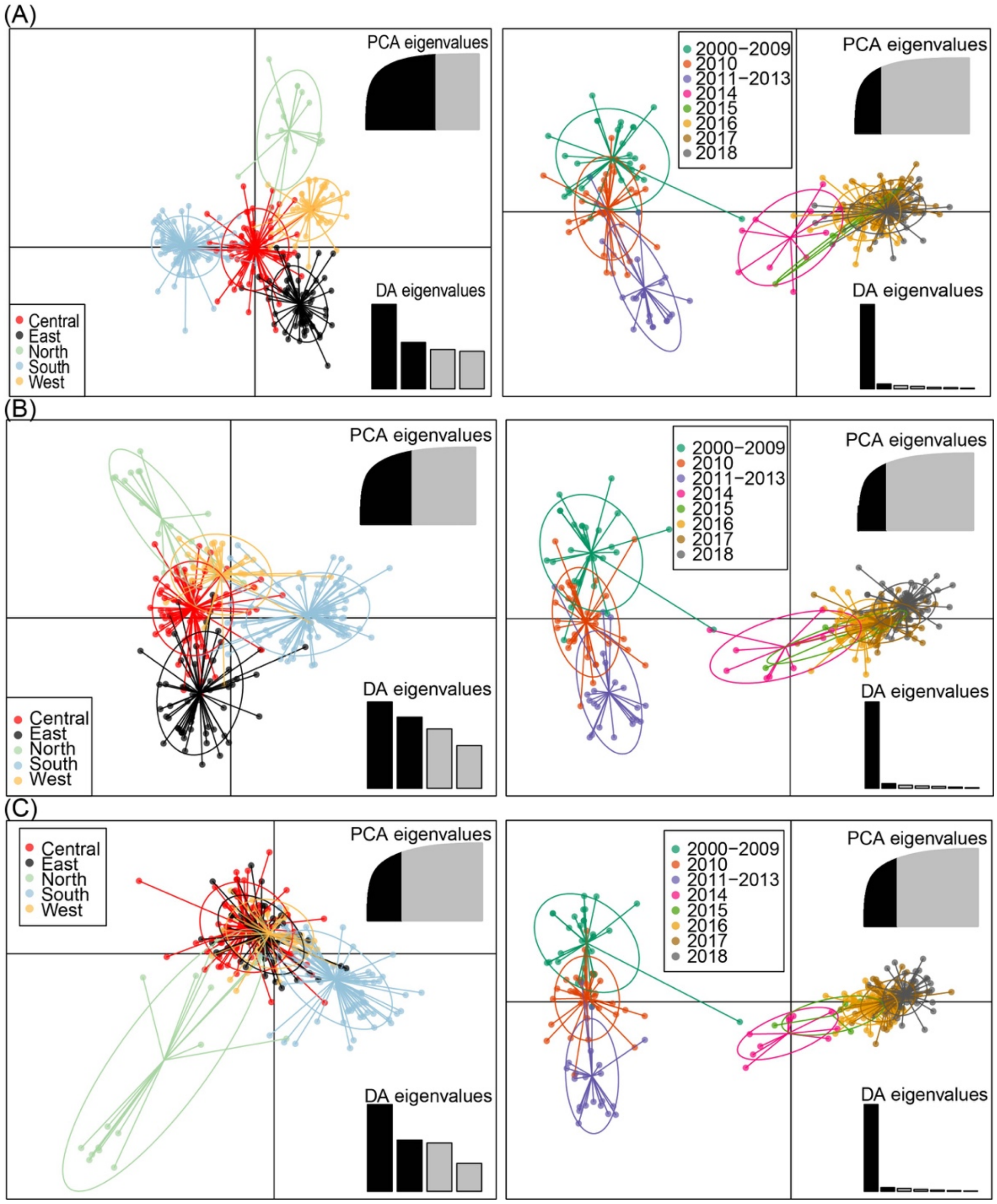

Fig S5. The subsequent scatterplot indicates the first two principal components of the DAPC of the cluster of 2014-2018. Eigenvalues of the analysis are displayed in the

inset. Groups are shown by different colors, and dots represent individual isolates

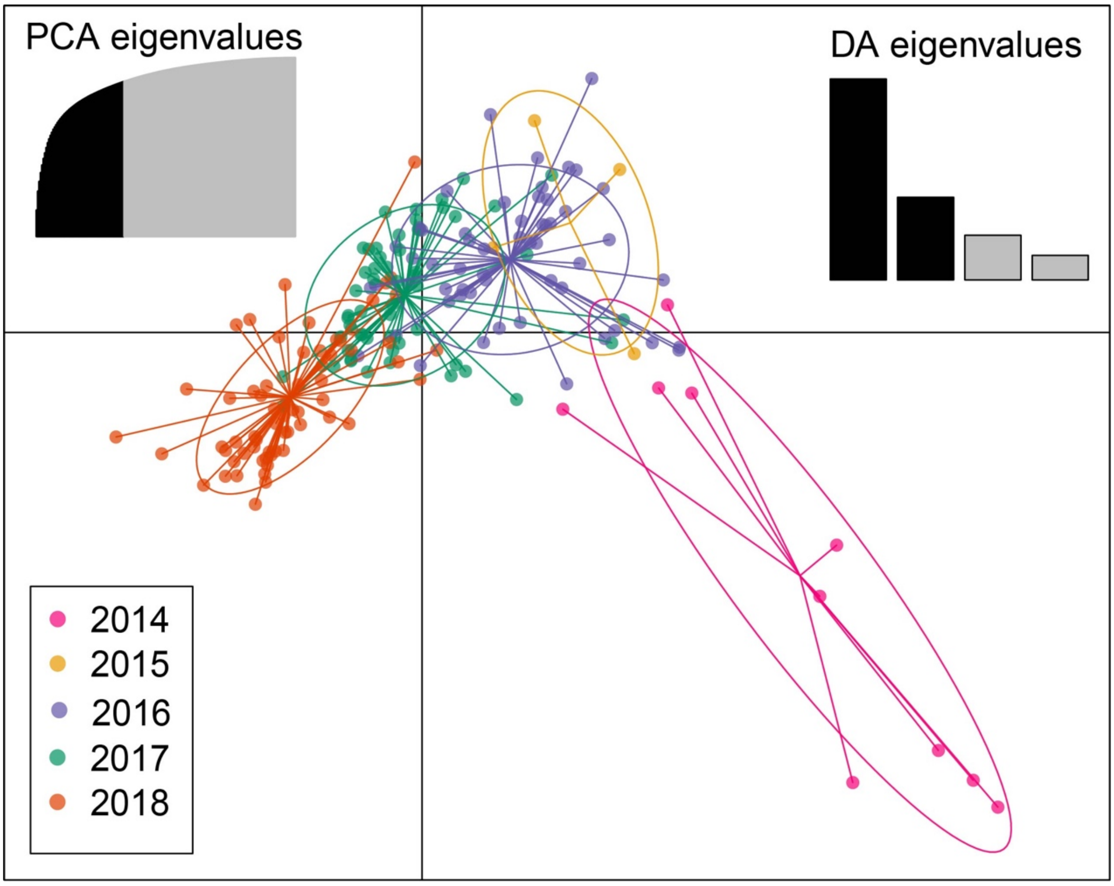

Fig S6. The neighbor-joining phylogenetic tree of the *VP1* and *3D* genomic region of CV-A16, respectively. The phylogenetic tree branches of strains are colored according

to the recombination forms (RFs). The scale bars represent the substitutions per site per year.

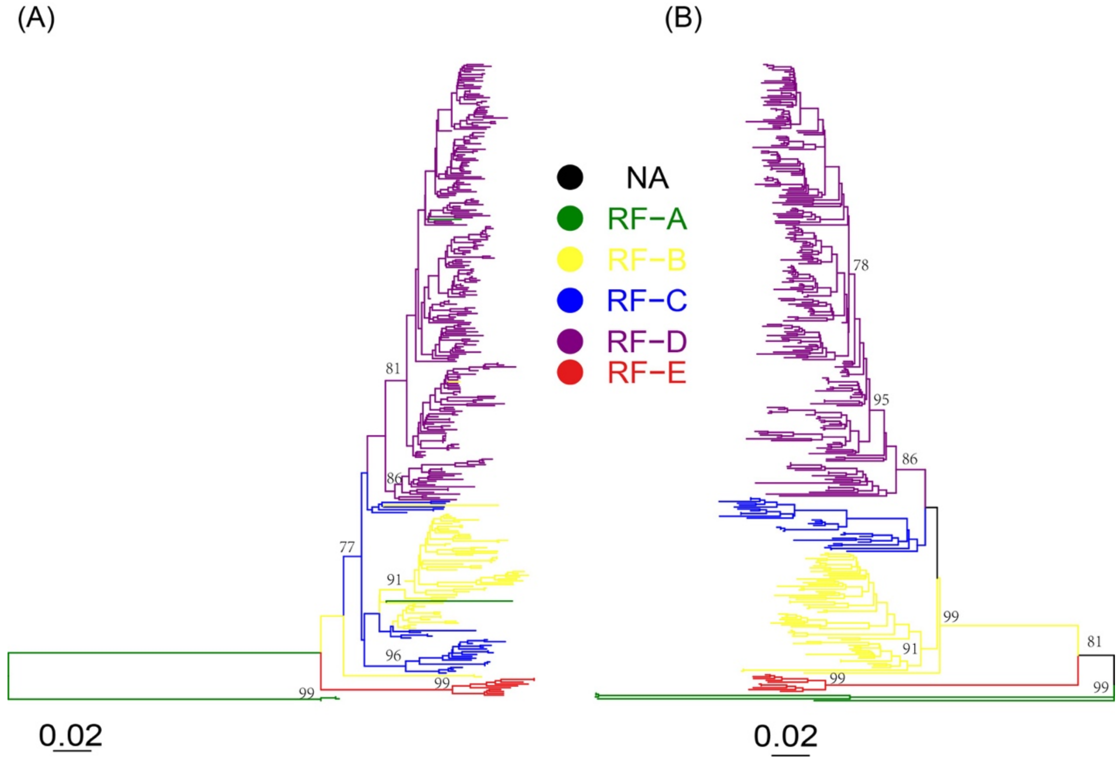

Fig S7. The midpoint-rooted maximum likelihood phylogenetic tree of 72 genomic sequences based on the *P1*, *P2*, and *P3* coding region, corresponding to the similarity

plot of Fig. 6. Group 1 was colored by red module, whereas the green and blue module represent the Groups 2 and 3, respectively.

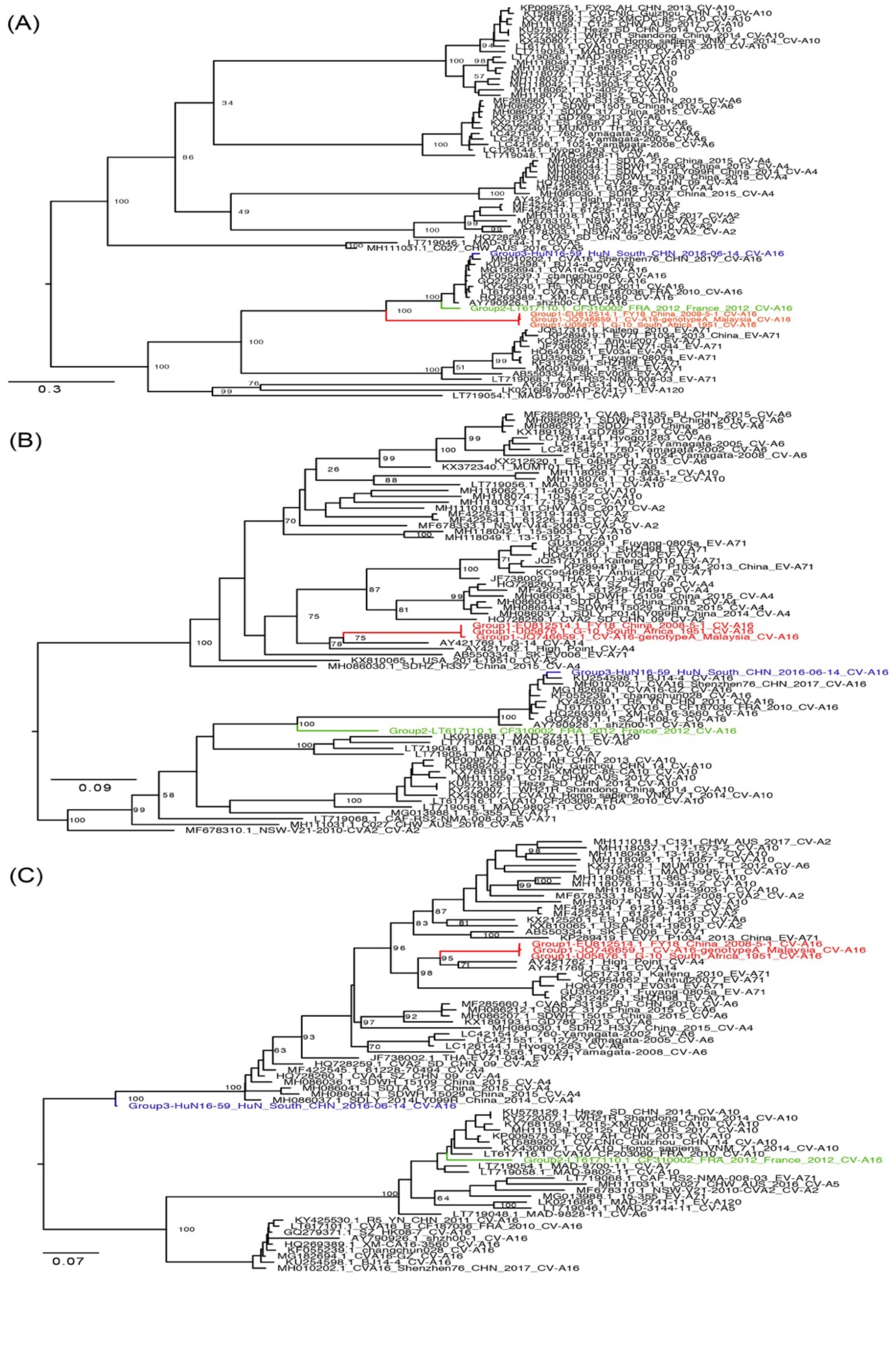

Table S1. The statistically supported migration rates of CV-A16 based on the *VP1* and *P1* coding regions.

From	To	*VP1*[a]			*P1*[a]		
		Mean migration rate	Indicator[b]	Bayes factor (BF)[c]	Mean migration rate	Indicator[b]	Bayes factor (BF)[c]
East	West	1.73	1	29353.79396	0.27	1	29353.79396
East	South	0.93	1	29353.79396	2.13	1	29353.79396
East	North	0.04	1	29353.79396	2.50	1	29353.79396
East	Central	0.66	1	29353.79396	0.53	1	29353.79396
North	Central	0.80	1	29353.79396	0.25	1	29353.79396
North	East	0.10	1	29353.79396	0.04	1	29353.79396
North	West	0.12	1	29353.79396	0.10	1	29353.79396
Central	East	NA	NA	NA	1.12	1	29353.79396
South	West	NA	NA	NA	0.65	1	29353.79396

West	East	0.27	1	29353.79396	NA	NA	NA
Central	North	0.70	1	29353.79396	NA	NA	NA

[a] The coding region of CV-A16 assessed for statistically supported migration rates.

[b] The posterior probability of observed a non-zero migration rate in the sampled trees.

[c] The indicator value > 0.5 and BF > 1000 of statistically supported migration rates are shown.

NA, no data available.

Table S2. Analysis of the different timescale structure of CV-A16 strains in China.

Statistic	Isolates	observed mean(95%HPD)	null mean(95%HPD)	significance
AI		2.03(1.64,2.61)	12.04(10.49,13.62)	0***
PS		17.36(16,18)	71.93(67.78,75.79)	0***
MC(2014-2018)	187	43.25(39,59)	7.75(5.58,10.59)	0.0099**
MC(2000-2013)	84	8.31(5,14)	2.82(2.06,3.95)	0.0099**

Analysis of the geographic structure of CV-A16 strains.

AI, association index; PS, parsimony score; MC, maximum monophyletic clade; HPD, highest probability density interval;

Significance thresholds:*,0.01<p<0.05;**,0.001<p<0.01;***,p<0.001

Table S3. The selection pressure of different datasets subsampled from mainland of China. For each dataset, the dN/dS and positively selection sites were calculated using MEME and SLAC methods.

Gene	Datasets	*VP1*		*P1**	
		dN/dS (SLAC)	Positively selection sites by MEME (amino acid position)	dN/dS (SLAC)	Positively selection sites by MEME (amino acid position)
Central	74	0.0255	145	0.0214	710
East	59	0.0313	NA	0.0216	NA
North	17	0.0125	NA	0.00962	NA
South	80	0.0223	145	0.0163	710
West	41	0.0157	NA	0.0114	52
2000-2013	84	0.0373	248	0.0336	813
2014-2018	187	0.0248	145	0.0163	NA
All	271	0.0294	145	0.0223	NA

NA, no data available.

* the total amino acid length of VP4, VP2 and VP3 was 565, which means that the length at VP1 adding 565 equal the length at P1 coding region.

Table S4. The primers used for amplification and sequencing.

Primer	Nucleotide position (nt)	Primer sequence (5’-3’)	Orientation
0001S48		GGGGACAAGTTTGTACAAAAAAGCAGGCTTTAAAACAGCTCTGG GGTT	Forward
CVA16-596Z	577-596	ACACGGACACCCAAAGTAGTCGGTTC	Reverse
EV/PCR-2	449-473	TCCGGCCCCTGAATGCGGCTAATCC	Forward
CVA16-1254Z	1235-1254	TGGAACTGAGCGTTTTGACC	Reverse
CVA16-1125F	1125	GATGCAACRGCAGTCGACAARCCC	Forward
CVA16-2095R	2095	CYGCAAACATGAATGTYACCTCCA	Reverse
CVA16-1790Y	1790-1809	ATTCTGCCAGGTTTCCATCC	Forward
CVA16-2800Z	2781-2800	CTGGGCATATCCCATCAAGT	Reverse
CVA16-2344F	2344	ATTGGTGCTCCYACTACAGCRTAT	Forward
CVA16-3345R	3345	CARCGTTGTTATCTTGTCTCTRCT	Reverse
CVA16-3292Y	3292-3311	GACATCAAATGCACCAGCAC	Forward
CVA16-4440Z	4421-4440	CGGTGTTTGCTCTTGAACTG	Reverse
CVA16-4342F	4342	TGTTTGGYAATGTGTCATATTTRG	Forward
CVA16-5389Y	5389	GCTCGGTCCTTGRACYGTGGCTGT	Reverse

CVA16-5204Y	5204-5223	CCAGAAACACCGACCAATGT	Forward
CVA16-6157Z	6138-6157	GTCAGGCTCGTGTAGGGTGT	Reverse
CVA16-5952Y	5952	GTGAACARGGAGAGATYCAATGGA	Forward
CVA16-6958Z	6958	ATARCTAGCYAACACATCATCTCC	Reverse
CVA16-6787Y	6787-6806	TTGTGTTCTTGGTGGAATGC	Forward
7500A		GGGGACCACTTTGTACAAGAAAGCTGGG$(T)_{24}$	Reverse

Table S5. The information of 346 coxsackievirus A16 (CV-A16) strains used in this analysis, including 49 isolates first reported in this study.

Strain name	GenBank accession No.	Isolation year	Countries	Origin
CVA16_B_CF310002_FRA_2012	LT617110.1	2012	France	GenBank
Tainan-5079-98	AF177911.1	NA	Taiwan	GenBank
shzh00-1	AY790926.1	2000	China	GenBank
shzh05-1	EU262658.1	2005	China	GenBank
FY18	EU812514.1	2008/5/1	China	GenBank
GZ08	FJ198212.1	2008/6/1	China	GenBank
SZ-HK08-3	GQ279368.1	2008/5/1	China	GenBank
SZ-HK08-7	GQ279371.1	2008/5/1	China	GenBank
XM-CA16-3560	HQ269389.1	2009/5/9	China	GenBank
KMM-08	HQ423141.1	2008/5/1	China	GenBank
THA-CA16-069	JF738004.1	2010/7/1	Thailand	GenBank
G20	JN590244.1	2010/5/24	China	GenBank
HN1662-HN-CHN-2010	JN674176.1	2010	China	GenBank
SH-CHN-2009	JQ034149.1	2010	China	GenBank
HQ09011181	JQ316639.1	2011/6/26	China	GenBank
Ningbo.CHN-028-2-2009	JQ354992.1	2009/4/2	China:	GenBank
CV-A16-genotypeA	JQ746659.1	NA	Malaysia	GenBank
PM-00033-07	JQ746660.1	2007	Malaysia	GenBank
PM-12284-99	JQ746661.1	1999	Malaysia	GenBank
PM-12727-99	JQ746662.1	1999	Malaysia	GenBank
PM-13884-97	JQ746663.1	1997	Malaysia	GenBank
PM-13998-00	JQ746664.1	2000	Malaysia	GenBank
PM-14660-97	JQ746665.1	1997	Malaysia	GenBank

PM-15765-00	JQ746666.1	2000	Malaysia	GenBank
PM-15922-00	JQ746667.1	2000	Malaysia	GenBank
PM-1651402-06	JQ746668.1	2006	Malaysia	GenBank
PM-16809-98	JQ746669.1	1998	Malaysia	GenBank
PM-16985-98	JQ746670.1	1998	Malaysia	GenBank
PM-1791021-07	JQ746671.1	2007	Malaysia	GenBank
PM-1795457-07	JQ746672.1	2007	Malaysia	GenBank
PM-22159-02	JQ746673.1	2002	Malaysia	GenBank
PM-22217-02	JQ746674.1	2002	Malaysia	GenBank
PM-23208-02	JQ746675.1	2002	Malaysia	GenBank
PM-31131-05	JQ746676.1	2005	Malaysia	GenBank
PM-31376-05	JQ746677.1	2005	Malaysia	GenBank
PM-35210-06	JQ746678.1	2006	Malaysia	GenBank
TS10-07	JX068827.1	2010/7/9	China	GenBank
BJ11-12	JX068828.1	2011/9/1	China	GenBank
TS10-08	JX068829.1	2010/8/1	China	GenBank
BJ11-03	JX068830.1	2011/3/1	China	GenBank
BJ-11-11	JX068831.1	2011/11/1	China	GenBank
BJ09-06	JX068832.1	2009/6/6	China	GenBank
BJ08-07	JX068833.1	2008/7/1	China	GenBank
BJCA08	JX481738.1	2008/2/11	China	GenBank
BJ1208	JX507808.1	NA	China	GenBank
Kor08-CVA16	JX839965.1	2008/6/1	South_Korea	GenBank
Wuhan0109-HuB-CHN-2011	JX986740.1	2011	China	GenBank
Wuhan0157-HuB-CHN-2011	JX986741.1	2011	China	GenBank
Wuhan0127-HuB-CHN-2011	JX986742.1	2011	China	GenBank

CA16-GD09-24	KC117317.1	2009	China:	GenBank
CA16-GD09-119	KC117318.1	2009	China	GenBank
G08	KC342228.1	NA	China	GenBank
YY157	KC507895.1	2010	China	GenBank
MAV	KC695830.1	NA	China	GenBank
XZ10-D-1	KC755228.1	2010	China	GenBank
ZJ10-73	KC755229.1	2010	China	GenBank
AH10-2	KC755230.1	2010	China	GenBank
AH10-12	KC755231.1	2010	China	GenBank
NJ10-31	KC755232.1	2010	China	GenBank
NJ10-75	KC755233.1	2010	China	GenBank
XZ10-C-1	KC755234.1	2010	China	GenBank
ZJ10-48	KC755235.1	2010	China	GenBank
CC024	KF055238.1	2010	China	GenBank
changchun028	KF055239.1	2010	China	GenBank
changchun029	KF055240.1	2010	China	GenBank
CC045	KF055241.1	2010	China	GenBank
changchun075	KF055242.1	2010	China	GenBank
CC090	KF055243.1	2010	China	GenBank
CC097	KF055244.1	2010	China	GenBank
CC163	KF055245.1	2010	China	GenBank
ZJ08-01	KF193620.1	2008	China	GenBank
SD09-05	KF193621.1	2009	China	GenBank
YN10-02	KF193622.1	2010	China	GenBank
HN11-03	KF193623.1	2011	China	GenBank
HN09-02	KF193624.1	2009	China	GenBank

GX10-01	KF193625.1	2010	China	GenBank
FJ10-03	KF193626.1	2010	China	GenBank
FJ09-02	KF193627.1	2009	China	GenBank
FJ09-01	KF193628.1	2009	China	GenBank
BJ10-03	KF193629.1	2010	China	GenBank
BJ10-01	KF193630.1	2010	China	GenBank
BJ10-02	KF193631.1	2010	China	GenBank
AH08-06	KF193632.1	2008	China	GenBank
CVA16-WIBP-P4-731	KF924762.1	2010/5/27	China	GenBank
DL16	KF991007.1	2012/5/18	China	GenBank
L23	KJ746492.1	2010	China	GenBank
CVA16-SZ29-CHN-2014	KM215267.1	2014/5/1	China	GenBank
Wh16	KM516102.1	2010/6/1	China	GenBank
2008-43-7	KP266573.1	2008/12/2	China	GenBank
CV-A16-P10-2013-China	KP289411.1	2013	China	GenBank
CV-A16-P1014-2013-China	KP289412.1	2013	China	GenBank
CV-A16-P187-2013-China	KP289413.1	2013	China	GenBank
CV-A16-P255-2013-China	KP289414.1	2013	China	GenBank
CV-A16-P301-2013-China	KP289415.1	2013	China	GenBank
CV-A16-P83-2013-China	KP289416.1	2013	China	GenBank
GDV126	KU163608.1	2010	China	GenBank
BJ14-3	KU254597.1	2014/7/9	China	GenBank
BJ14-4	KU254598.1	2014/7/9	China	GenBank
CA16-193	KU854873.1	2008	China	GenBank
CA16-194	KX056216.1	2008	China	GenBank
ensh01-CHN-12	KX058533.1	2012	China	GenBank

SiICRC04-TH-2011	KX372333.1	2011/8/10	Thailand	GenBank
SiICRC05-TH-2011	KX372334.1	2011/8/15	Thailand	GenBank
SiICRC06-TH-2011	KX372335.1	2011/8/16	Thailand	GenBank
SiICRC01-TH-2012	KX372336.1	2012/8/8	Thailand	GenBank
SiICRC02-TH-2012	KX372337.1	2012/12/1	Thailand	GenBank
SiICRC03-TH-2012	KX372338.1	2012/12/1	Thailand	GenBank
SiICRC01-TH-2014	KX372339.1	2014/7/8	Thailand	GenBank
CA16-196	KX580041.1	2008/8/7	China	GenBank
CVA16-Shenzhen36-CHN-2014	KX595291.1	2014/5/1	China	GenBank
CVA16-Shenzhen73-CHN-2014	KX595292.1	2014/6/1	China	GenBank
CVA16-Shenzhen74-CHN-2014	KX595293.1	2014/6/1	China	GenBank
CVA16-Shenzhen79-CHN-2014	KX595294.1	2014/7/1	China	GenBank
CVA16-Shenzhen179-CHN-2014	KX595295.1	2014/4/1	China	GenBank
393	KY014077.1	2008/8/28	China	GenBank
K168-8	KY088084.1	2010/8/6	China	GenBank
K11-YN-CHN-2011	KY425528.1	2011/6/6	China	GenBank
V1-YN-CHN-2015	KY425529.1	2015/6/13	China	GenBank
R5-YN-CHN-2011	KY425530.1	2011/6/2	China	GenBank
K34-YN-CHN-2011	KY425531.1	2011/5/1	China	GenBank
R68-YN-CHN-2009	KY425532.1	2009/5/24	China	GenBank
R35-YN-CHN-2012	KY425533.1	2012/6/23	China	GenBank
R34-YN-CHN-2012	KY425534.1	2012/6/23	China	GenBank
R37-YN-CHN-2013	KY425535.1	2013/10/14	China	GenBank
V37-YN-CHN-2014	KY425536.1	2014/6/24	China	GenBank
R141-YN-CHN-2009	KY425537.1	2009/6/24	China	GenBank
R254-YN-CHN-2010	KY425538.1	2010/5/18	China	GenBank

R255-YN-CHN-2010	KY425539.1	2010/5/18	China	GenBank
V86-YN-CHN-2015	KY425540.1	2015/6/19	China	GenBank
CV-A16-A01-BLR-IN	KY792576.1	2012/10/1	India	GenBank
CV-A16-A02-BLR-IN	KY792577.1	2012/10/1	India	GenBank
CV-A16-A06-BLR-IN	KY792578.1	2013/7/1	India	GenBank
CV-A16-A10-BLR-IN	KY792579.1	2013/7/1	India	GenBank
CV-A16-A13-BLR-IN	KY792580.1	2013/7/1	India	GenBank
CV-A16-A122-BLR-IN	KY792581.1	2015/6/1	India	GenBank
CV-A16-A128-BLR-IN	KY792582.1	2015/6/1	India	GenBank
CV-A16-M02-BLR-IN	KY792583.1	2013/4/1	India	GenBank
CV-A16-M69-BLR-IN	KY792584.1	2015/10/1	India	GenBank
CVA16_B_RP80_AUT_2003	LT617091.1	2003	Austria	GenBank
CVA16_B_BER53-2_DEU_2003	LT617092.1	2003	Germany	GenBank
CVA16_B_BUD22_HUN_2008	LT617093.1	2008	Hungary	GenBank
CVA16_B_BUD25_HUN_2008	LT617094.1	2008	Hungary	GenBank
CVA16_B_STU7_DEU_2009	LT617096.1	2009	Germany	GenBank
CVA16_B_STU4_DEU_2010	LT617097.1	2010	Germany	GenBank
CVA16_B_CF145057_FRA_2010	LT617098.1	2010	France	GenBank
CVA16_B_CF160074_FRA_2010	LT617099.1	2010	France	GenBank
CVA16_B_CF166109_FRA_2010	LT617100.1	2010	France	GenBank
CVA16_B_CF187036_FRA_2010	LT617101.1	2010	France	GenBank
CVA16_B_CF279014_FRA_2010	LT617102.1	2010	France	GenBank
CVA16_B_CF312044_FRA_2010	LT617103.1	2010	France	GenBank
CVA16_B_CF223065_FRA_2011	LT617104.1	2011	France	GenBank
CVA16_C_CF350028_FRA_2011	LT617105.1	2011	France	GenBank
CVA16_C_CF172083_FRA_2012	LT617106.1	2012	France	GenBank

CVA16_C_CF178025_FRA_2012	LT617107.1	2012	France	GenBank
CVA16_C_CF178036_FRA_2012	LT617108.1	2012	France	GenBank
CVA16_C_CF193053_FRA_2012	LT617109.1	2012	France	GenBank
CVA16_C_PAR155055_FRA_2014	LT617111.1	2014	France	GenBank
CVA16_C_MET171023_FRA_2014	LT617112.1	2014	France	GenBank
CVA16_C_LYO171046_FRA_2014	LT617113.1	2014	France	GenBank
CVA16_C_PAR181046_FRA_2014	LT617114.1	2014	France	GenBank
CVA16_C_PAR190033_FRA_2014	LT617115.1	2014	France	GenBank
USA-CT-2016-19518	MF189180.1	2016/11/16	USA	GenBank
ZJ6	MF434051.1	2008/8/28	China	GenBank
HF146-SD-CHN-2008	MG450666.1	2008	China	GenBank
TA271-Shandong-China-2015	MG674827.1	2015/7/18	China	GenBank
CVA16-Shenzhen500-CHN-2014	MH010198.1	2014/12/1	China	GenBank
CVA16-Shenzhen469-CHN-2015	MH010199.1	2015/10/1	China	GenBank
CVA16-Shenzhen87-CHN-2016	MH010200.1	2016/4/1	China	GenBank
CVA16-Shenzhen289-CHN-2016	MH010201.1	2016/6/1	China	GenBank
CVA16-Shenzhen76-CHN-2017	MH010202.1	2017/3/1	China	GenBank
CVA16-Shenzhen169-CHN-2017	MH010203.1	2017/5/1	China	GenBank
CVA16-Shenzhen174-CHN-2017	MH010204.1	2017/5/1	China	GenBank
CVA16-Shenzhen189-CHN-2017	MH010205.1	2017/5/1	China	GenBank
CVA16-Shenzhen220-CHN-2017	MH010206.1	2017/5/1	China	GenBank
C028-CHW-AUS-2016	MH111067.1	2016/4/22	Australia	GenBank
C105-CHW-AUS-2016	MH111068.1	2017/1/18	Australia	GenBank
C123-CHW-AUS-2016	MH111070.1	2017/2/17	Australia	GenBank
C138-CHW-AUS-2016	MH111071.1	2016/12/14	Australia	GenBank
C151-CHW-AUS-2016	MH111072.1	2017/4/16	Australia	GenBank

G-10	U05876.1	1951	South_Africa	GenBank
AH17-18/AH/East/CHN/2017-02-12	MT211988	2017/2/12	China	this study
AH18-25/AH/East/CHN/2018-04-26	MT211989	2018/4/26	China	this study
BJ16-68/BJ/Central/CHN/2016-08-01	MT211990	2016/8/1	China	this study
BJ17-14/BJ/Central/CHN/2017-02-15	MT211991	2017/2/15	China	this study
BJ18-58/BJ/Central/CHN/2018-08-20	MT211992	2018/8/20	China	this study
CQ16-104/CQ/West/CHN/2016-01-13	MT211993	2016/1/13	China	this study
CQ17-38/CQ/West/CHN/2017-04-12	MT211994	2017/4/12	China	this study
CQ18-3/CQ/West/CHN/2018-06-28	MT211995	2018/6/28	China	this study
GD16-105/GD/South/CHN/2016-06-17	MT211996	2016/6/17	China	this study
GD17-81/GD/South/CHN/2017-06-01	MT211997	2017/6/1	China	this study
GD18-104/GD/South/CHN/2018-08-14	MT211998	2018/8/14	China	this study
GS16-110/GS/West/CHN/2016-05-30	MT211999	2016/5/30	China	this study
GS17-636/GS/West/CHN/2017-06-15	MT212000	2017/6/15	China	this study
GS17-658/GS/West/CHN/2017-06-15	MT212001	2017/6/15	China	this study
GS18-179/GS/West/CHN/2018-05-14	MT212002	2018/5/14	China	this study
GZ16-QN035/GZ/South/CHN/2016-06-30	MT212003	2016/6/30	China	this study
HAN17-66/HaN/South/CHN/2017-12-11	MT212004	2017/12/11	China	this study
HAN18-8/HaN/South/CHN/2018-02-28	MT212005	2018/2/28	China	this study
HB16-54015/HB/Central/CHN/2016-01-15	MT212006	2016/1/15	China	this study
HB17-54371/HB/Central/CHN/2017-07-07	MT212007	2017/7/7	China	this study
HB18-20/HB/Central/CHN/2018-05-16	MT212008	2018/5/16	China	this study
HEN17-108/HeN/Central/CHN/2017-06-30	MT212009	2017/6/30	China	this study
HeN18-400/HeN/Central/CHN/2018-07-11	MT212010	2018/7/11	China	this study
HLJ16-HH2016033/HLJ/North/CHN/2016-09-19	MT212011	2016/9/19	China	this study
HLJ18-16/HLJ/North/CHN/2018-08-05	MT212012	2018/8/5	China	this study

HuN16-75/HuN/South/CHN/2016-05-09	MT212013	2016/5/9	China	this study
HUN17-33/HuN/South/CHN/2017-08-07	MT212014	2017/8/7	China	this study
HuN18-5/HuN/South/CHN/2018-02-26	MT212015	2018/2/26	China	this study
JL18-97/JL/North/CHN/2018-08-05	MT212016	2018/8/5	China	this study
JX16-126/JX/East/CHN/2016-12-27	MT212017	2016/12/27	China	this study
JX18-30/JX/East/CHN/2018-03-26	MT212018	2018/3/26	China	this study
LN16-23-12/LN/North/CHN/2016-07-11	MT212019	2016/7/11	China	this study
QH16-8/QH/West/CHN/2016-07-07	MT212020	2016/7/7	China	this study
QH17-104/QH/West/CHN/2017-10-13	MT212021	2017/10/13	China	this study
QH18-5/QH/West/CHN/2018-05-12	MT212022	2018/5/12	China	this study
SAX17-50/SaX/Central/CHN/2017-09-16	MT212023	2017/9/16	China	this study
SD16-101/SD/East/CHN/2016-07-22	MT212024	2016/7/22	China	this study
SX17-286/SX/Central/CHN/2017-06-16	MT212025	2017/6/16	China	this study
TJ16-7/TJ/Central/CHN/2016-02-29	MT212026	2016/2/29	China	this study
TJ17-36/TJ/Central/CHN/2017-03-13	MT212027	2017/3/13	China	this study
TJ18-63/TJ/Central/CHN/2018-05-28	MT212028	2018/5/28	China	this study
XJ17-212/XJ/West/CHN/2017-10-04	MT212029	2017/10/4	China	this study
XJ18-019/XJ/West/CHN/2018-04-09	MT212030	2018/4/9	China	this study
YN16-253/YN/South/CHN/2016-05-17	MT212031	2016/5/17	China	this study
YN17-J29/YN/South/CHN/2017-02-07	MT212032	2017/2/7	China	this study
YN18-A67/YN/South/CHN/2018-03-14	MT212033	2018/3/14	China	this study
ZJ16-14/ZJ/East/CHN/2016-03-30	MT212034	2016/3/30	China	this study
ZJ17-91/ZJ/East/CHN/2017-07-02	MT212035	2017/7/2	China	this study
ZJ18-24/ZJ/East/CHN/2018-01-04	MT212036	2018/1/4	China	this study

Table S6. The model selection of BEAST. According parameters were listed, including the path sampling (PS) and stepping stone sampling (SS) values.

Molecular clock model	Coalescent tree prior	ESS	PS	SS
strict clock	GMRF	>200	-43879.9	-43887.4
strict clock	constant size	>200	-43846.4	-43854.1
strict clock	exponential growth	>200	-43790.6	-43801.1
strict clock	BSP	>200	-43772.4	-43788.7
strict clock	EBSP	>200	NA	NA
strict clock	SkyGrid	<200	-43847.7	-43857.1
Uncorrelated exponential relaxed clock	GMRF	>200	-43809.1	-43819.7
Uncorrelated exponential relaxed clock	constant size	<200	-43821.2	-43832.4
Uncorrelated exponential relaxed clock	exponential growth	<200	-43787.7	-43795.3
Uncorrelated exponential relaxed clock	BSP	<200	-43757.5	-43773.6
Uncorrelated exponential relaxed clock	EBSP	<200	NA	NA
Uncorrelated exponential relaxed clock	SkyGrid	<200	-43811.5	-43820.1
Uncorrelated lognormal relaxed clock	GMRF	>200	-43733.4	-43748.2
Uncorrelated lognormal relaxed clock	constant size	>200	-43738.8	-43752.8
Uncorrelated lognormal relaxed clock	exponential growth	<200	-43722.1	-43732.1
Uncorrelated lognormal relaxed clock	BSP	<200	-43668.2	-43687.4
Uncorrelated lognormal relaxed clock	EBSP	<200	NA	NA
Uncorrelated lognormal relaxed clock	SkyGrid	<200	-43742.4	-43756.9
